# RNA-recognition motif in Matrin-3 mediates neurodegeneration through interaction with hnRNPM

**DOI:** 10.1186/s40478-020-01021-5

**Published:** 2020-08-18

**Authors:** Nandini Ramesh, Sukhleen Kour, Eric N. Anderson, Dhivyaa Rajasundaram, Udai Bhan Pandey

**Affiliations:** 1 Department of Pediatrics, Children’s Hospital of Pittsburgh, University of Pittsburgh Medical Center, Pittsburgh, PA 15224 USA; 2 Department of Human Genetics, University of Pittsburgh, School of Public Health, Pittsburgh, PA USA; 3 Department of Pediatrics, Division of Health Informatics, Children’s Hospital of Pittsburgh , Pittsburgh, PA USA

**Keywords:** ALS, Myopathy, Matrin-3, hnRNPM, RNA-binding proteins, *Drosophila*

## Abstract

**Background:**

Amyotrophic lateral sclerosis (ALS) is an adult-onset, fatal neurodegenerative disease characterized by progressive loss of upper and lower motor neurons. While pathogenic mutations in the DNA/RNA-binding protein Matrin-3 (MATR3) are linked to ALS and distal myopathy, the molecular mechanisms underlying MATR3-mediated neuromuscular degeneration remain unclear.

**Methods:**

We generated *Drosophila* lines with transgenic insertion of human MATR3 wildtype, disease-associated variants F115C and S85C, and deletion variants in functional domains, ΔRRM1, ΔRRM2, ΔZNF1 and ΔZNF2. We utilized genetic, behavioral and biochemical tools for comprehensive characterization of our models in vivo and in vitro. Additionally, we employed in silico approaches to find transcriptomic targets of MATR3 and hnRNPM from publicly available eCLIP datasets.

**Results:**

We found that targeted expression of MATR3 in *Drosophila* muscles or motor neurons shorten lifespan and produces progressive motor defects, muscle degeneration and atrophy. Strikingly, deletion of its RNA-recognition motif (RRM2) mitigates MATR3 toxicity. We identified rump, the *Drosophila* homolog of human RNA-binding protein hnRNPM, as a modifier of mutant MATR3 toxicity in vivo. Interestingly, hnRNPM physically and functionally interacts with MATR3 in an RNA-dependent manner in mammalian cells. Furthermore, common RNA targets of MATR3 and hnRNPM converge in biological processes important for neuronal health and survival.

**Conclusions:**

We propose a model of MATR3-mediated neuromuscular degeneration governed by its RNA-binding domains and modulated by interaction with splicing factor hnRNPM.

**Electronic supplementary material:**

The online version of this article (10.1186/s40478-020-01021-5) contains supplementary material, which is available to authorized users.

## Background

Amyotrophic lateral sclerosis (ALS) is a fatal neurodegenerative disorder that leads to progressive loss of upper and lower motor neurons [1]. ALS pathogenesis is increasingly linked to mutations in genes encoding RNA-binding proteins [2–4]. Indeed, in post-mortem brain and spinal cord tissues, many such RNA-binding proteins are sequestered into pathological aggregates in the cytoplasm [5]. Preclinical models implicate dysregulation of RNA metabolism as an underlying mechanism linking RNA-binding proteins to ALS pathogenesis [6–9].

Mutations in Matrin-3 (MATR3), a DNA/RNA-binding nuclear matrix protein, were initially discovered in North American patients with familial ALS [10]. Additional ALS-causing mutations in *MATR3* were later identified in other cohorts; currently 13 nonsynonymous point mutations are implicated in familial and sporadic ALS [11–14]. One such mutation, p.S85C, is also linked to inherited distal myopathy with vocal and pharyngeal weakness (VCPDM) followed by neurogenic changes [10]. Interestingly, the same mutation is also found in patients with only myopathic symptoms [15–17]. Thus, *MATR3* is among the family of genes including *VCP*, *HNRNPA1*, *HNRNPA2B1* and *SQSTM1* that cause “multisystem proteinopathy” associated with either one or a combination of ALS/frontotemporal dementia (FTD), VCPDM and Paget’s disease of bone [18]. Additionally, MATR3-positive inclusions have been discovered in C9orf72-mediated- [10] and sporadic ALS [19], underscoring the link between MATR3 and neurodegenerative pathology.

**Fig. 1:**
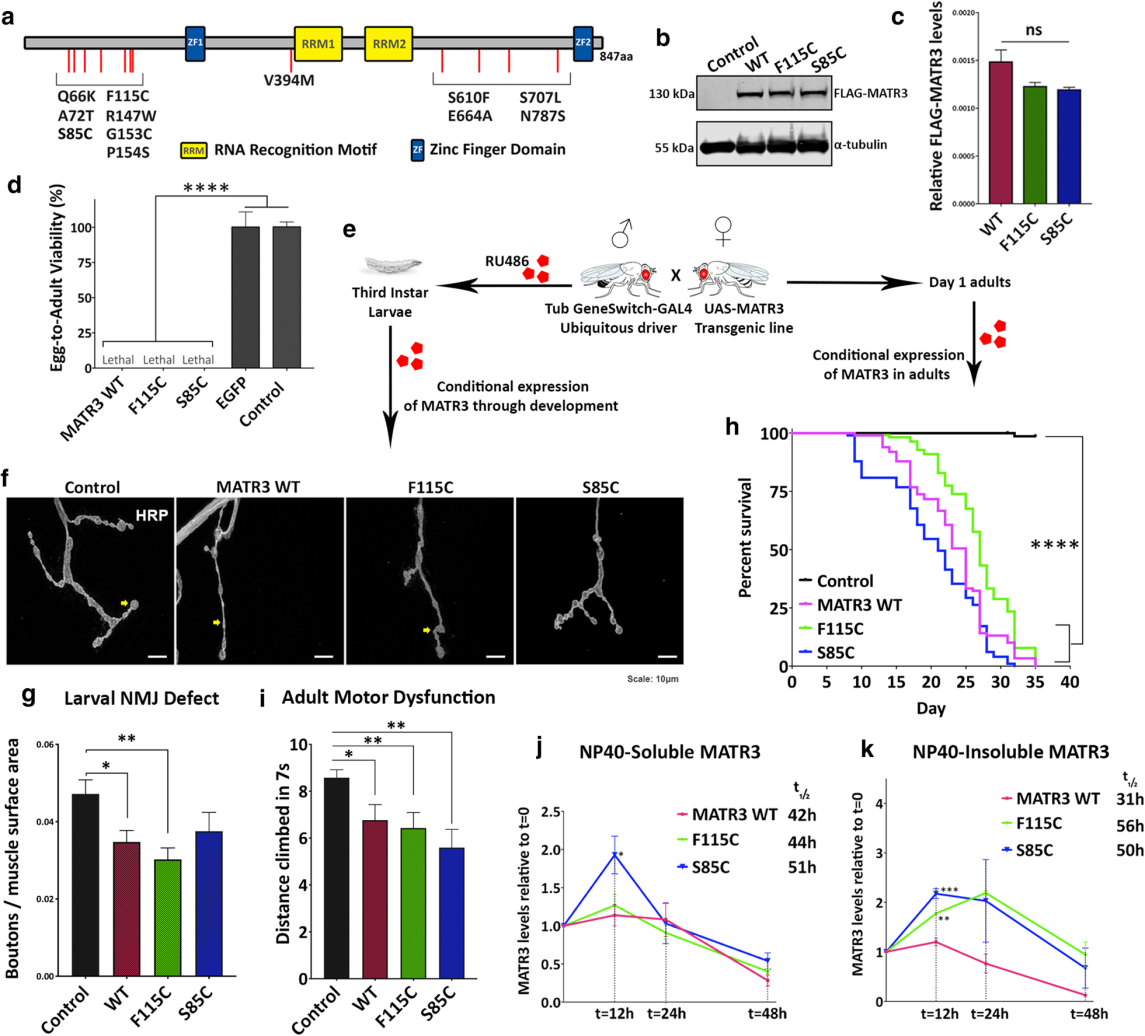
Ubiquitous expression of MATR3 is toxic in *Drosophila.* **a** Schematic diagram of MATR3 protein domain architecture, consisting of two tandem RNA-recognition motifs (RRM1 and RRM2) and two Zinc Finger motifs (ZF1 and ZF2). ALS-associated mutations in MATR3 are spread across intrinsically disordered regions of the protein. **b** Immunoblot showing transgenic expression of UAS-FLAG-MATR3 wildtype and mutants – F115C and S85C. **c** Quantification of replicate western blots to confirm equal transgenic expression levels due to site-specific integration. α-tubulin is used as loading control (n = 3 per group; *One*-*way ANOVA*). **d** Constitutive ubiquitous expression of MATR3 wildtype and mutants, driven by Tub-Gal4, is toxic in *Drosophila* leading to complete lethality. Quantification of egg-to-adult viability showed that control (driver-alone) and transgenic UAS-EGFP expression had no effect on viability (n = 3, *One*-*way ANOVA*). **e** Schematic of conditional expression of MATR3 during development and in adults using the inducible driver, TubGS-Gal4, that is activated by RU486. **f** Representative immunofluorescence images of third-instar larval neuromuscular junction (NMJ) immunostained for presynaptic marker, HRP. Yellow arrows point to the synaptic boutons. **g** Quantification of boutons, normalized to surface area, showed reduced number of boutons in MATR3-expressing larvae (n = 6-8; *One*-*way ANOVA*). **h** Kaplan–Meier survival curve of adults ubiquitously expressing MATR3 showing statistically-significant reduction in longevity of MATR3-expressing flies compared to driver-alone control flies (n = 80; *Log*-*Rank Mantel*-*Cox test*) **i** Quantification of motor dysfunction in day-12 adults expressing MATR3, induced on day-1. (n = 25-40, *One*-*way ANOVA*). **j** Quantification of NP40-soluble and **k** NP40-insoluble MATR3 degradation in vivo by a pulse-chase assay at t = 0, 12, 24 and 48 h following inhibition of transgene production (n = 3-4 per group, *One*-*way ANOVA*). Statistical significance shown for F115C and S85C groups compared to WT at same time point. NP40-soluble and insoluble MATR3 mutants have a higher half-life compared to MATR3 WT. Error bars indicate S.E.M. *p < 0.05; **p < 0.01; ****p < 0.0001

As an essential nuclear matrix protein, MATR3 maintains the fibrogranular network and has functions in various aspects of RNA metabolism including alternative splicing [20–22], maintaining mRNA stability [23], transcription [23, 24] and mRNA export [25, 26]. Additionally, MATR3 interacts with SFPQ and NONO to mediate the DNA-damage response [27]. The protein is composed mostly of intrinsically disordered regions (IDRs), except for 4 functional domains: two tandem RNA-recognition motifs and two C2H2-type zinc finger domains (Fig. 1a). Mutations associated with ALS do not fall within any known functional domain, however most pathogenic mutations are clustered in the protein N-terminus (Fig. 1a). The MATR3 N-terminus region forms droplet-like structures in cells [28], indicating that this region is required for mediating physiological liquid–liquid phase separation of MATR3. However, the physiological role of MATR3 phase separation and its relevance to disease pathogenesis is yet to determined.

In vitro and in vivo studies indicate that MATR3 dosage is key to neuronal survival. In NSC-34 motor neuron-like cells, ectopic expression of wild type MATR3 and its ALS-associated mutations impedes nuclear mRNA export, including export of *FUS* and *TDP*-*43* mRNA [26]. Either overexpression or knockdown of MATR3 in rat cortical neurons is sufficient to negatively impact their survival [29]. In mice, homozygous knockout of *Matr3* is embryonic lethal [30]. On the other hand, transgenic overexpression of the MATR3 ALS-linked F115C mutant leads to development of severe myopathic degeneration and paralysis in an age-dependent manner [31]. The crucial remaining question is how mutations in MATR3 lead to disease pathogenesis of ALS/FTD and distal myopathy.

To address this, we developed a transgenic *Drosophila* model expressing either the human wild type or mutant (F115C and S85C) MATR3. Our model exhibits significant neuromuscular degeneration, enabling us to dissect basic biology of MATR3 in an in vivo model system. We show that MATR3-mediated toxicity in vivo is regulated by its RNA-binding domains, suggesting that mutations in MATR3 could lead to disease pathogenesis through its RNA-binding functions. Furthermore, we show that MATR3 genetically and physically interacts with the splicing factor hnRNPM, which is required for mutant MATR3 to exert toxicity in *Drosophila*.

## Materials and methods

### Plasmids

FLAG-MATR3 WT, ΔRRM1, ΔRRM2, ΔZNF1 and ΔZNF2 in pCMV Tag2B vector were a gift from Yossi Shiloh (Addgene plasmid #32880, 32881. 32882, 32883, 32884). pCMV-Tag2B FLAG-MATR3 S85C and pCMV-Tag2B FLAG-MATR3 F115C plasmids were generated by acquiring IDT gene fragments (gBlock) corresponding to S85C and F115C sequences and sub-cloning it into pCMV-Tag2B MATR3 WT plasmid between ScaI-EcoRI restriction sites. For generating *Drosophila* lines, FLAG-MATR3 constructs were cloned into pUASTaTTB vector between NotI-XhoI restriction sites. All sequences were verified by Sanger sequencing. The gBlock fragment and primer sequences are listed in Additional file 1: Table S5. pT7-V5-SBP-C1-HshnRNPM was a gift from Elisa Izaurralde (Addgene plasmid # 64924).

### Generation and maintenance of *Drosophila* lines

UAS-MATR3 lines were generated by site-specific insertion of the transgene in a *w*^*1118*^ background by BestGene Inc. The detailed list of other lines used in this study and their respective sources are outlined in Additional file 2: Table S6. All *Drosophila* stocks were cultured on standard dextrose media on a 12-hour light/dark cycle.

### Egg-to-adult viability assay

UAS-MATR3 lines were crossed with ubiquitous driver, Tubulin-Gal4/TM3, on standard media at 28 °C. The crosses were set up in triplicate. The expected progeny from the cross is 50% of flies carrying the TM3 balancer, i.e. flies not expressing MATR3, and 50% of flies not carrying TM3 balancer, i.e. flies that are expressing MATR3. Egg-to-adult viability was measured as a percentage of observed # flies/expected # flies.

### Larval neuromuscular junction (NMJ) analysis

UAS-MATR3 lines were crossed with inducible driver Tubulin-GS-Gal4, at 28 °C on standard media mixed with 10 µM final concentration RU486 (Cayman Chemicals) for inducing transgene expression. Third-instar wandering larvae were picked for NMJ analysis. For immunofluorescence, the larvae were rinsed in PBS (Lonza 17-512F) and dissected along the dorsal midline to expose the NMJs and performed as outlined in “Immunofluorescence” section of Methods. Confocal images were acquired in Z-stacks using Nikon A1 microscope at 60x (oil) magnification. NMJs innervating Muscle 4 at hemi-segments A3-A4 were used for analyzing synaptic bouton quantities. For each NMJ, number of synaptic boutons per NMJ was normalized to surface area of innervating muscle. ImageJ (NIH) was used for quantification of bouton number and NMJ area.

### Adult motor dysfunction

For conditional ubiquitous expression, UAS-MATR3 lines were crossed with inducible driver, TubGS-Gal4. Day 1 adults from the F1 progeny were collected every 24 h and moved to standard media mixed with 20 mM RU486. For constitutive expression in specific tissues, UAS-MATR3 lines were crossed with either muscle-specific driver, MHC-Gal4, or motor neuron-specific driver, OK371-Gal4. The F1 adults were cultured at 28 °C.

Locomotion was assessed using the RING assay, as previously described [32] with a few modifications. Briefly, flies were transferred, without anesthetization, into plastic vials and placed in the RING apparatus. The vials were tapped down against the bench and the climbing was recorded on video. Quantifications were performed manually by a third party in a blinded manner.

### *Drosophila* sectioning and H&E staining

For assessment of muscle morphology, UAS-MATR3 flies were crossed with muscle-specific MHC-Gal4 driver. The F1 progeny adults were aged for 30 days when the thorax was dissected out and fixed in Davidson’s fixative, modified (Electron Microscopy Sciences 64133-10). For assessment of retinal morphology, UAS-MATR3 flies were crossed with eye-specific GMR-Gal4 driver. The heads were resected from F1 adults and fixed using Davidson’s fixative. Sectioning and H&E staining was done at Excalibur Pathologies. Light microscopy images were acquired in using Leica DM5500 at 10X magnification and quantification of muscle surface area was performed using ImageJ (NIH).

### Pulse-chase assay

UAS-MATR3 flies were crossed with inducible ubiquitous driver, TubGS-Gal4. F1 adults were starved overnight (placed in vials with Kimwipe soaked in water) and then placed on standard media mixed with 20 mM RU486 for 12 h to induce transgene expression. The flies were then transferred non-RU486 media to stop transgene production and start the “chase” experiment. Protein degradation was chased by flash-freezing flies at 0, 12, 24 and 48 h after transferring flies to non-RU486 media and start of chase, followed by preparation of fly lysates and SDS-PAGE as described below. For determining half-life, time taken for protein levels to reach 0.5 relative to starting protein amount (t = 0) was interpolated from the curve. If relative protein levels did not reach 0.5 at 48 h, the graph was extrapolated to determine half-life.

### Preparation of fly lysates and SDS-page

*Drosophila* tissue was flash frozen on dry ice and crushed using pestles. For preparation of standard lysate, crushed tissue was incubated in RIPA buffer: 150 mM NaCl, 1% NP40, 0.1% SDS, 1% sodium deoxycholate, 50 mM NaF, 2 mM EDTA, 1 mM DTT, 2.5 mM Na orthovanadate, 1 × protease inhibitor cocktail (Roche 11836170001), pH 7.4. The samples were sonicated in an ultrasonic bath and centrifuged down at 12,000×*g* for 10 min. The supernatant was boiled in 1X NuPage™ LDS-Sample buffer (Invitrogen NP0007) at 95 °C for 5 min.

For soluble-insoluble fractionation, flash frozen tissue was crushed and resuspended in NP40 lysis buffer: 0.5% NP40, 10 mM Tris HCl pH 7.8, 10 mM EDTA, 150 mM NaCl, 2.5 mM Na orthovanadate, 1x protease inhibitor cocktail (Roche 11836170001). The lysate was sonicated in an ultrasonic bath and centrifuged at 21000xg for 25 min. The supernatant (soluble fraction) was collected and boiled in 1X NuPage™ LDS-Sample buffer (Invitrogen, NP0007) at 95 °C for 5 min. The pellet was washed by resuspending in Washing buffer: 50 mM Tris HCl pH 7.4, 150 mM NaCl, and centrifuged at 21000xg for 5 min. The pellet was then resuspended in resolubilization buffer: 50 mM Tris HCl pH 6.8, 5% SDS, 10% glycerol followed by sonication and centrifugation at 12000xg for 10 min. The supernatant (insoluble fraction) was collected and boiled in 1X NuPage™ LDS-Sample buffer (Invitrogen, NP0007) at 95 °C for 5 min.

SDS-PAGE was performed using 3-8% NuPage™ Tris–Acetate gels (Invitrogen) and the separated proteins were transferred onto nitrocellulose membranes using the iBlot2 system (Life Technologies 13120134).

### Co-immunoprecipitation (Co-IP)

HEK293T cells were cultured in 10-cm dishes and co-transfected with FLAG-MATR3 and V5-HNRNPM as described below. 24 h post-transfection, cells were trypsinized and pelleted down. Nuclear lysates were extracted from the cell pellets using the NE-PER kit according manufacturer’s instructions (Thermo Scientific 78833) and incubated with 8 µl/ml FLAG antibody (Sigma F1804) overnight at 4 °C. The lysates were incubated with Dynabeads™ Protein G superparamagnetic beads (Invitrogen 10004D) for 4 h at 4 °C. For RNase A treatment, the samples were treated with 1 mg/ml RNaseA at this stage for 1 h at 4 °C. The beads were washed and purified using MagnaRack™ (Invitrogen CS15000). The immunoprecipitated samples were resuspended in 1X NuPage™ LDS-Sample buffer (Invitrogen NP0007) and boiled at 95 °C for 5 min, followed by SDS-PAGE and Immunoblotting.

### RNA-immunoprecipitation (RNA-IP)

HEK293T cells were cultured in 10-cm dishes and co-transfected with V5-HNRNPM along with either FLAG-MATR3 WT, FLAG-MATR3 F115C or FLAG-MATR3 S85C. 48 h-post transfection, cells were harvested and resuspended in lysis buffer (100 mM KCl, 5 mM MgCl_2_, 10 mM HEPES pH 7, 0.5% NP-40, 1 mM DTT, 100 U/µl RNase Inhibitor, 2 mM vanadyl ribonucleoside complex). Cells were lysed by vortexing and incubation on ice for 40 min followed by sonication. Cell debris was pelleted by centrifugation at 16000xg for 10 min. 50 µl of supernatant was set aside as “Input”. The remaining lysate was divided equally and treated separately with FLAG antibody (8 µg/ml, Sigma F1804), V5 antibody (8 µg/ml, Invitrogen R960-25) and negative antibody control (-ve Ab) and incubated overnight with rotation at 4 °C. The samples were then incubated with Dynabeads™ Protein G superparamagnetic beads (Invitrogen 10004D) for 4 h at 4 °C. The beads were washed with was buffer (50 mM Tris–HCL pH 7.4, 150 mM NaCl, 1 mM MgCl_2_, 0.05% NP-40, 20 mM EDTA pH 8.0, 1 mM DTT, 50 U/µl RNase inhibitor, 2 mM ribovanadyl nucleoside complex). Following washes, the beads were resuspended in wash buffer. 20ug proteinase K and 1% final concentration SDS were added to each sample including “Input” followed by incubation at 65 °C for 1 h. The beads were purified using MagnaRack™ (Invitrogen CS15000) followed by RNA extraction using phenol-choloroform-isoamyl alcohol.

cDNA synthesis was performed using the Applied Biosystems High capacity cDNA Reverse Transcription kit (4368814) and was subsequently run using the Bio-Rad iQ™Supermix (170-8862) on a 96-well plate (Applied Biosystems, #4306737) on Applied Biosystems StepOnePlus Real-Time PCR system. Ct values from FLAG-immunoprecipitation and V5-immunoprecipitation from each group were normalized to their respective “Input” Ct value (ΔCt) and normalized again to “-ve Ab control” (ΔΔCt). Fold change differences were evaluated as previously described [33] and statistical analysis was performed on Prism Graphpad. Primer and probe sequences used for quantitative real time-PCR are listed in Additional file 3: Table S7.

### Cell culture and transfections

HEK293T cells (ATCC^®^ CRL-3216™) and C2C12 cells (ATCC^®^ CRL-1772™) were cultured in Advanced DMEM supplemented with 10% FBS and 1% Glutamax and grown at 37 °C and 5% CO_2_. HEK293T cells were transiently transfected using Lipofectamine 3000 (Invitrogen L3000001) and used 24 h after transfection. C2C12 cells were transiently transfected using Turbofect (Invitrogen) and fixed for immunofluorescence at 48 h post-transfection.

### Immunoblotting

Nitrocellulose membranes were blocked in blocking buffer: 5% milk (BLOT- QuickBlocker^™^ EMD Millipore WB57) in TBST followed by overnight incubation with primary antibody at 4 °C. Membranes were washed with TBST and incubated with secondary antibody for 1 h at room temperature, followed by washed with TBST. The membranes were imaged on Odyssey^®^ CLx (LI-COR Biosciences) and quantification of bands was performed using Image Studio™ (LI-COR Biosciences).

Primary and secondary antibodies were prepared in blocking buffer.

*Primary antibodies*: Mouse anti-FLAG (1:1000; Sigma F1804); Rabbit anti-MATR3 (1:4000; Abcam ab151714); Mouse anti-V5 (1:1000; Invitrogen R960-25); Mouse anti- α tubulin (1:8000; Sigma T5168)

*Secondary antibodies*: Goat anti-mouse Dylight 680 (1:10000; LI-COR 925-68070); Goat anti-rabbit Dylight 680 (1:10000; Invitrogen 35568); Goat anti-mouse Dylight 800 (1:10000; Invitrogen SA5-10176); Goat anti-rabbit Dylight 800 (1:10000; Invitrogen SA5-35571)

### Immunofluorescence

Dissected *Drosophila* tissues or C2C12 cells grown on coverslips were rinsed in PBS (Lonza 17-512F) and fixed in 4% paraformaldehyde (Sigma P6148) for 20 min at room temperature. Following fixation, the samples were washed four times (×10 min) in PBS and blocked with blocking buffer: 5% normal goat serum (NGS; Abcam AB7681) in PBS with 0.1% TritonX-100 (PBST). The samples were incubated with primary antibody overnight at 4 °C, washed four times (x10 min) with 0.1% PBST, incubated with secondary antibody for 2 h at room temperature followed by 0.1% PBST washes. Samples were mounted onto slides using either ProLong^®^ Gold Antifade mounting reagent (Invitrogen P36930) or Fluoroshield (Sigma F6057).

Primary and secondary antibodies were prepared in blocking buffer.

*Primary antibodies*: Cy3-conjugated anti-HRP (1:100, Jackson ImmunoResearch 123-165-021); Rabbit anti-FLAG (1:500, Sigma F7425); Mouse anti-hnRNPM 2A6 (1:100; Santa Cruz sc-20001); Rabbit anti-MATR3 (1:500; Abcam ab151714)

*Secondary antibodies*: Goat anti-rabbit Alexa Fluor 488 (1:1000; Invitrogen A11008); Goat anti-mouse Alexa Fluor 546 (1:1000; Invitrogen A11030)

### Quantitative Real Time-PCR

RNA was extracted from the heads of flies ubiquitously expressing MATR3 using PureLink RNA Mini Kit (Invitrogen 12183018A0) following the manufacturer’s instructions. cDNA synthesis was performed using the iScript Select cDNA Synthesis Kit (BioRad; 170-8897) and was subsequently run using the Bio-Rad iQ™Supermix (170-8862) on a 96-well plate (Applied Biosystems, #4306737) on Applied Biosystems StepOnePlus Real-Time PCR system. Primer pairs and probes were designed for each target of interest and housekeeping control α-tubulin using Integrated DNA Technologies PrimeTime qPCR Assay (www.idtdna.com). The comparative Ct method was used for analyzing the fold change differences as previously described [33] and statistical analysis was performed on Prism Graphpad. Primer and probe sequences used for quantitative real time-PCR are listed in Additional file 3: Table S7.

### eCLIP data analysis

The previously published [34] datasets for MATR3 eCLIP and hnRNPM eCLIP and corresponding input controls from HepG2 and K562 cell lines were acquired from the ENCODE portal. The pipeline from the standard operating procedure (SOP) published on the ENCODE website was followed [34]. In brief, FASTQ files were adapter-trimmed, mapped, and PCR duplicates removed. The uniquely mapped reads termed as usable reads were used for downstream analysis. As a second processing pipeline, CLIP BAM files were normalized over the input and fold-change enrichment within enriched peak regions was estimated (*p* value for enrichment was calculated by Yates’ Chi Square test or Fisher Exact Test). Enriched peaks were then annotated to GRCh38 version of the genome using HOMER [35] and filtered based on significance (p-value < 0.05) and fold-change (≥ 4) for further analysis (Additional file 4: Table S2). Motif discovery was performed using the findMotifsGenome.pl wrapper from HOMER v4.11 [35]. De novo motif finding was done using default genomic positions as the background and -len 8, and -rna as parameters.

To generate read density plots around MATR3 peaks in each cell type, the midpoint of significantly enriched peaks on the transcripts bound by both MATR3 and hnRNPM were identified. The distribution of hnRNPM read density around the midpoint of the enriched MATR3 eCLIP peak was plotted using a window of -400nt to +400nt. Similarly, read density plots were generated for distribution of MATR3 read density around the midpoint of enriched hnRNPM eCLIP peaks ± 400 nt.

### Gene ontology enrichment

List of unique annotated transcripts bound by MATR3 and hnRNPM at 3′-UTR, 5′-UTR, intron and exons were obtained from eCLIP analysis and compared for shared targets (Additional file 5: Table S3). Gene ontology analysis was performed using ToppGene Suite (ToppFun). A list of enriched “GO:Biological Processes” and “GO:Diseases” were generated by ToppFun. The top-20 unique biological processes were plotted (Fig. 7c, d) and the corresponding gene list for each biological process was listed (Additional file 6: Table S4).

### Statistical analysis

All statistical analyses were performed on Graphpad Prism6.

## Results

### Ubiquitous expression of *MATR3* in *Drosophila* leads to neuromuscular junction defects followed by motor dysfunction

To investigate the role of MATR3 in an in vivo model system, we generated transgenic *Drosophila* lines with site-specific integration of either wild type (WT) human *MATR3* or *MATR3* with ALS-associated mutations Ser85Cys (S85C) and Phe115Cys (F115C) with an N-terminal FLAG tag. We confirmed similar transgene expression levels between groups before further analysis (Fig. 1b, c). We then utilized the well-established UAS/GAL4 ectopic expression system to systematically assess the effect of MATR3 expression in target tissues. Constitutive ubiquitous expression of WT or mutant MATR3 in *Drosophila* caused developmental lethality, leading to complete loss of egg-to-adult viability (Fig. 1d). In contrast, neither the non-transgenic control (standard *w*^*1118*^ strain crossed with Tub-Gal4 driver) nor ubiquitous expression of an irrelevant transgene, *EGFP*, caused any lethality, confirming that the phenotype was exclusively due to MATR3 expression (Fig. 1d).

To assess our model for specific motor neuron defects, we utilized the GeneSwitch (GS) expression system induced by a mild dose of RU486 for controlled conditional expression of MATR3 through development to larval stages (Fig. 1e). We labeled the third-instar larval neuromuscular junction (NMJ), a well-established model for investigating neurodegeneration, for the presynaptic marker HRP to assess synaptic bouton morphology. Expression of MATR3 WT and F115C, but not S85C, reduced the number of synaptic boutons (normalized to muscle area) in the NMJ compared to controls (Fig. 1f, g). To determine if ubiquitous expression of MATR3 results in motor dysfunction among adult animals, we used the inducible expression system to bypass expression in developmental stages and instead conditionally express MATR3 in adults, induced by RU486 (Fig. 1e). Consistent with neuromuscular junction defects in larvae, conditional expression of MATR3 in adults resulted in statistically significant reduction in their locomotion ability compared to controls (Fig. 1i). Additionally, these flies had a significantly shortened lifespan compared to controls (Fig. 1h). We observed a strong nuclear WT and mutant MATR3 signal with a granular expression pattern (Additional file 7: Fig S1) in the *Drosophila* ventral nerve chord (VNC), suggesting that pathogenic mutations do not perturb the subcellular distribution of MATR3 protein in our model.

We next performed a pulse-chase experiment to analyze MATR3 degradation kinetics in vivo. We briefly induced MATR3 expression using RU486, then chased protein turnover using immunoblotting. Since MATR3 associates with both detergent (NP40)-soluble and insoluble fractions, we analyzed protein stability in both fractions. NP40-soluble wild type and F115C MATR3 exhibited similar degradation kinetics (Fig. 1j), with the F115C mutant protein exhibiting a slightly longer half-life (t_1/2_ = 44 h) compared to WT (t_1/2_ = 42 h). The NP40-soluble S85C mutant, on the other hand, accumulated for the first 12 h then gradually degraded, resulting in an overall increase in half-life of the protein (t_1/2_ = 51 h) (Fig. 1j). However, NP40-insoluble MATR3 exhibited different kinetics: insoluble F115C and S85C mutants accumulated in the first 12 h and thus took longer to degrade compared to the WT protein, resulting in overall increase in half-life of insoluble species of F115C (t_1/2_ = 56 h) and S85C (t_1/2_ = 50 h) compared to that of WT (t_1/2_ = 36 h) (Fig. 1k). This suggests that pathogenic mutations in MATR3 result in decreased protein turn-over, leading to more stable insoluble forms of the protein that might disrupt its functions.

### Muscles exhibit higher vulnerability to the ALS/myopathy-linked MATR3 S85C mutation

**Fig. 2:**
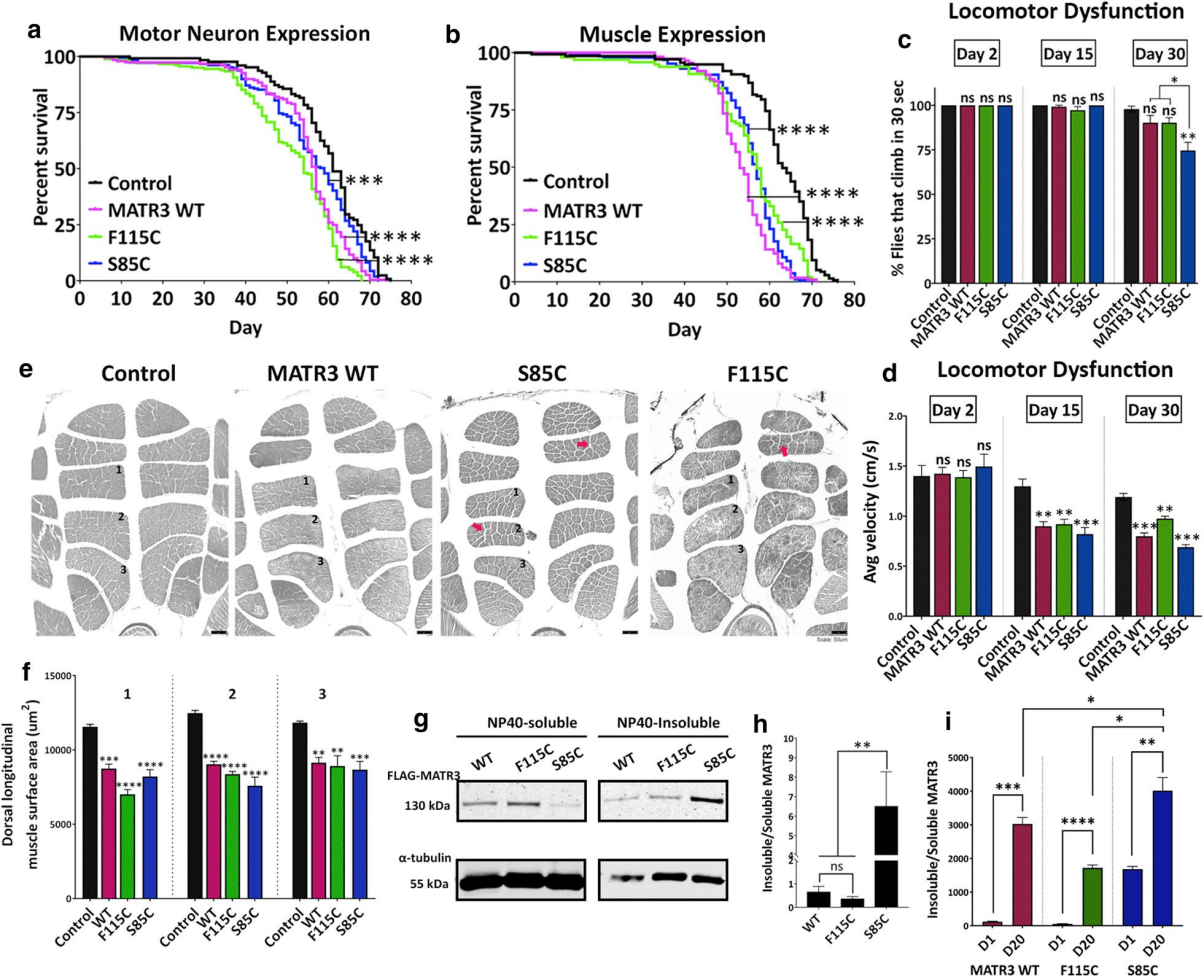
Tissue-specific expression of MATR3 leads to progressive degeneration in motor neurons and muscles. **a** Kaplan–Meier survival curve in flies constitutively expressing MATR3 in motor neurons, driven by OK371-Gal4, and **b** in muscles, driven by MHC-Gal4. Expression of MATR3 WT and mutants in either tissue moderately, but significantly, reduced longevity of the flies compared to driver-alone control (n = 100; *Log*-*Rank Mantel*-*Cox test*). **c** Quantification of the percent flies that can climb and **d** average velocity at which they climb at day-2, day-15 and day-30 of adult lifespan (n = 50; *One*-*way ANOVA*), **e** H&E stained indirect flight muscles in transverse thoracic cross-sections in 30-day old flies expressing MATR3 in muscles. Red arrows indicate enlarged gaps between muscle fibers in the S85C and F115C-expressing flies indicating muscle degeneration. **f** Quantification of the cross-sectional area of dorsal-longitudinal muscle (DLM) segments 1, 2 and 3. DLMs of MATR3-expressing flies showed lower cross-sectional area compared to driver-alone control, with F115C and S85C flies exhibiting more pronounced atrophy (n = 4 hemi-segments per group; *One*-*way ANOVA*). **g** Immunoblot showing the NP40-soluble and NP40-insoluble fractions of MATR3 in *Drosophila* thorax lysates from flies expressing MATR3 in muscles. **h** Quantification of insoluble/soluble MATR3 protein fraction. S85C mutant protein is significantly more enriched in the insoluble fraction (n = 4-6 per group; *One*-*way ANOVA*). α-tubulin is used as loading control. **i** Quantification of insoluble/soluble MATR3 protein fraction at day-1 and day-20 of adult fly lifespan. MATR3 WT, F115C and S85C are significantly more enriched in the insoluble fraction at day-20 compared to day-1. MATR3-S85C at day-20 is significantly more enriched in the insoluble fraction compared to WT and F115C at day-20 (n = 4-6 per group; *One*-*way ANOVA*). Error bars indicate S.E.M. *p < 0.05; **p < 0.01; ***p < 0.001; ****p < 0.0001

To dissect the tissue-specific roles of MATR3, we expressed MATR3 in disease-relevant tissues, including motor neurons and muscles. While not as potent as in the ubiquitous expression paradigm, expression of MATR3 in either motor neurons (Fig. 2a, Additional file 8: Fig S2A) or muscles (Fig. 2b, Additional file 8: Fig S2B) significantly shortened the lifespan compared to controls, indicating that tissue-specific MATR3 expression results in neuronal and muscular degeneration, respectively. Furthermore, expression of MATR3 in *Drosophila* eyes did not cause external or internal eye degeneration, even when aged for 30 days (Additional file 9: Fig S3). These finding suggest that MATR3-mediated toxicity is not systemic, and that motor neurons and muscles might be specifically susceptible to MATR3 expression.

To examine whether reduced longevity is supported by concurrent motor dysfunction, we tested the locomotion abilities of flies expressing MATR3 in muscles. At day 2, MATR3-expressing flies do not exhibit any obvious motor deficit (Fig. 2c, d). However, at day 15 of the adult lifespan, while MATR3 flies retained their climbing ability, the speed at which they climbed was significantly impaired compared to controls (Fig. 2d). Interestingly, the motor dysfunction in S85C mutant flies was exacerbated in an age-dependent manner; by day 30, approximately 25% of animals entirely lost their climbing ability (Fig. 2c). Among the flies that did climb, their climbing velocity remained significantly lower compared to that of controls (Fig. 2d). This suggests that while reduced lifespan induced by tissue-specific MATR3 expression occurs at a later age, climbing defects start to manifest as early as day-15, suggesting a slow and progressive deterioration.

To investigate if these locomotion defects are caused by morphological defects in the muscles, we performed hematoxylin and eosin (H&E) staining on cross-sections of the dorsal longitudinal muscles (DLMs) in the thorax. DLMs in MATR3-expressing flies appeared smaller compared to those of controls, most likely due to muscle atrophy in these animals (Fig. 2e, f). Complementary to motor dysfunction, the atrophy phenotype was particularly severe in S85C mutant flies, which exhibited enlarged gaps between muscle fibers, indicating muscle degeneration (Fig. 2e). F115C mutant flies showed similar degeneration, but to a lesser degree than the S85C mutant flies (Fig. 2e). This finding demonstrates that muscle-targeted expression of MATR3 leads to adult-onset degeneration that is progressive in the pathogenic S85C mutant.

We next utilized this model to identify biochemical alterations caused by MATR3 mutations that lead to acquisition of toxic properties, particularly solubility of MATR3 in NP40-containing buffers. In *Drosophila* muscles, MATR3 WT and F115C mutant proteins were distributed almost evenly between the soluble and insoluble fractions (Fig. 2g, h). However, the S85C mutation drastically reduced the solubility of the protein, with most of the protein accumulating in the insoluble fraction (Fig. 2g, h). Next, we took advantage of the age-dependent onset of toxicity in the muscle-expression paradigm to assess for any potential correlation between toxicity and solubility. Soluble-insoluble fractionation of MATR3 in *Drosophila* muscles in day 1-old and day 20-old flies showed significantly higher enrichment of MATR3 protein in the insoluble fraction at day 20 compared to day 1 in both WT and mutant groups (Fig. 2i). Interestingly, at day 20, relative insolubility of S85C mutant remained higher to that of WT and F115C, suggesting that S85C mutant is possibly more prone to become insoluble (Fig. 2i).

### RNA-binding domains of MATR3 drive toxicity in vivo

**Fig. 3:**
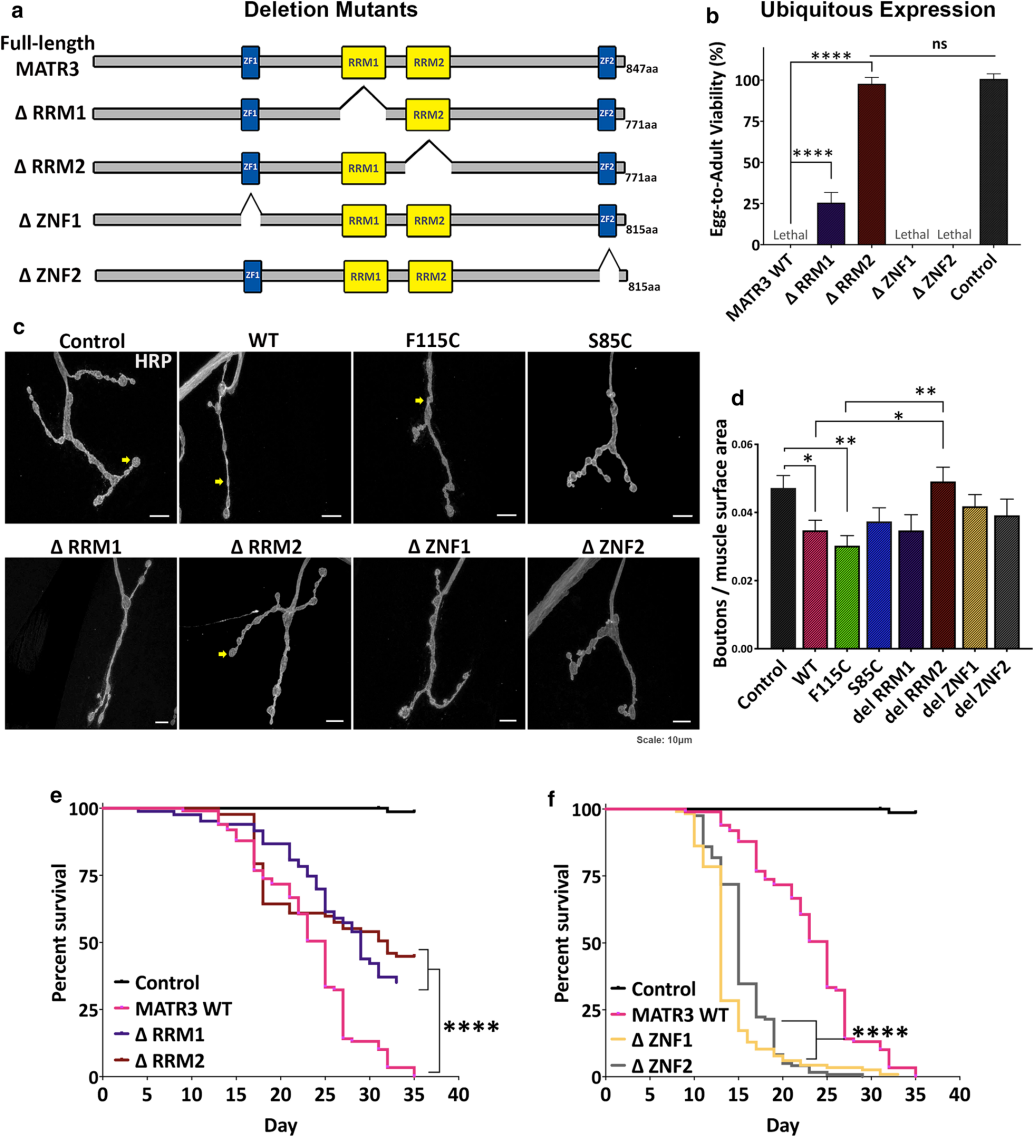
MATR3 toxicity is mediated through its RNA-binding domains. **a** Schematic diagram of MATR3 protein domain architecture in deletion mutant-transgenic flies, where each of the known functional domains are deleted. **b** Quantification of egg-to-adult viability in flies ubiquitously expressing MATR3 deletion mutants, driven by Tub-Gal4 driver. Constitutive ubiquitous expression of MATR3 deletion mutants showed partial rescue by ΔRRM1 and complete rescue by ΔRRM2 (n = 3, *One*-*way ANOVA*). **c** Representative immunofluorescence images of third-instar larval neuromuscular junction (NMJ) immunostained for presynaptic marker, HRP. Yellow arrows point to the synaptic boutons. **d** Quantification of number of synaptic boutons, normalized to surface area, showed that ΔRRM2 restored number of synaptic boutons back to near-control levels *(*n = 8, *One*-*way ANOVA*). **e** Kaplan–Meier survival curve of adults ubiquitously expressing MATR3 deletion mutants under the conditional driver. Both ΔRRM1 and ΔRRM2 significantly extended MATR3 lifespan, while **f** ΔZNF1 and ΔZNF2 further reduced MATR3 lifespan *(*n = 100, *Log*-*rank Mantel*-*Cox test*) Error bars indicate S.E.M. *p < 0.05; **p < 0.01; ****p < 0.0001

We sought to determine which functional domain(s) of MATR3 is responsible for mediating toxicity. We generated transgenic MATR3 lines with deletion mutations in each of the four functional domains: ΔRRM1, ΔRRM2, ΔZNF1 and ΔZNF2 (Fig. 3a). When ubiquitously expressed, deletion of the RRM2 domain completely rescued developmental lethality, yielding adults expressing MATR3 (Fig. 3b). RRM1 deletion also partially rescued developmental toxicity (Fig. 3b). However, deletion of either of the zinc-finger domains (ZNF1 or ZNF2) did not suppress toxicity (Fig. 3b). To determine if RRM1/2 deletion similarly modulates the NMJ defect, we induced conditional MATR3 expression with RU486 and labeled the third instar larval NMJs for HRP. Importantly, RRM2 deletion was sufficient to strongly rescue the NMJ defects caused by MATR3 (Fig. 3c), restoring the number of synaptic boutons to near-control levels (Fig. 3d). We then moved to an adult expression paradigm to evaluate if RRM2 deletion retains its rescue ability. In adults, deletion of RRM1 and RRM2, independently, significantly extended the lifespans of flies expressing MATR3 (Fig. 3e). However, unlike in developmental toxicity, ΔRRM2 adults retain some toxicity, with a shorter lifespan compared to controls (Fig. 3e). Taken together, adult longevity analyses suggest that MATR3 toxicity may be mediated by both RRM1 and RRM2 RNA-binding domains. ZNF1/2 deletion, on the other hand, exacerbated toxicity in the adults by significantly shortening their lifespan (Fig. 3f).

**Fig. 4:**
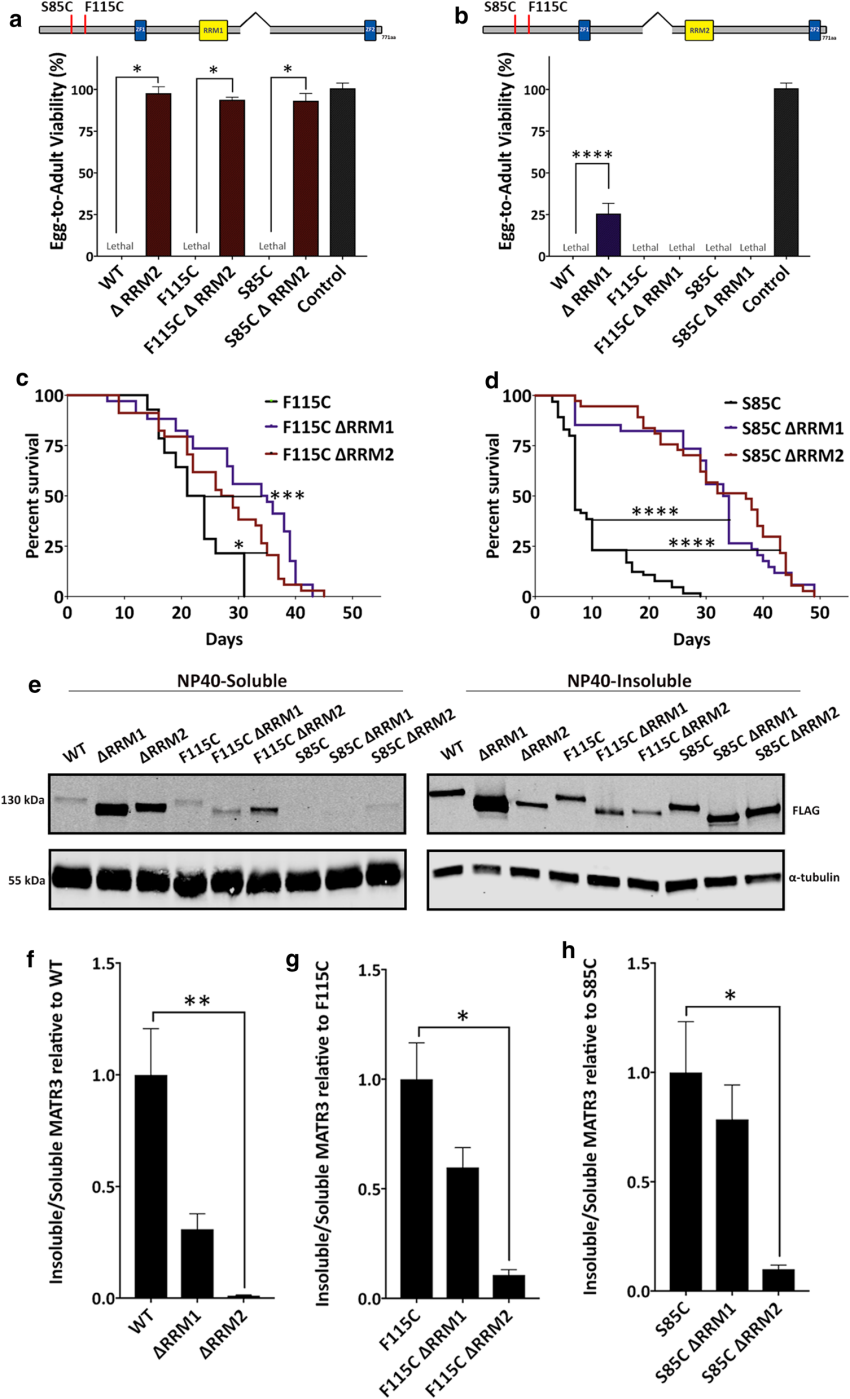
RRM2 deletion recues mutant MATR3 toxicity in vivo. **a** Quantification of egg-to-adult viability in flies expressing double mutants: F115C ΔRRM2 and S85C ΔRRM2. Constitutive ubiquitous expression of ΔRRM2 in the mutant background completely rescued mutant MATR3-mediated developmental toxicity (n = 3, *One*-*way ANOVA*). **b** Quantification of egg-to-adult viability in flies expressing double mutants: F115C ΔRRM1 and S85C ΔRRM1. Constitutive ubiquitous expression of ΔRRM1 in the mutant background is not sufficient to rescue mutant MATR3-mediated developmental toxicity (n = 3, *One*-*way ANOVA*). **c** Kaplan–Meier survival curve of adults ubiquitously expressing MATR3 deletion mutants in F115C background and **d** S85C background under conditional driver (TubGS-Gal4). ΔRRM1 and ΔRRM2 significantly increased longevity of adults expressing the MATR3 mutants *(*n = 50, *Log*-*rank Mantel*-*Cox test*). **e** Immunoblot of NP40-soluble and NP40-insoluble fractions of MATR3 from lysates of flies expressing full-length WT and mutant MATR3, and corresponding deletion mutants. α-tubulin is used as loading control. **f** Quantification of the insoluble/soluble fractions of full-length MATR3 and deletion mutants, ΔRRM1 and ΔRRM2, in the background of WT, **g** F115C and **h** S85C. Deletion of RRM2 significantly increased the solubility of MATR3 WT, F115C and S85C (n = 4 per group; *One*-*way ANOVA*) Error bars indicate S.E.M. *p < 0.05; **p < 0.01; ***p < 0.001; ****p < 0.0001

These observations prompted us to examine the role of RRM1 and RRM2 domains in mediating toxicities associated with disease-causing mutations in MATR3. To test this, we generated mutant transgenic lines including F115C-ΔRRM1, F115C-ΔRRM2, S85C-ΔRRM1 and S85C-ΔRRM2. RRM2 deletion was equally successful in rescuing developmental toxicity in flies expressing pathogenic mutant MATR3 (Fig. 4a). Interestingly, ΔRRM1, while mildly protective on its own, did not have any rescue effect on mutant MATR3 developmental toxicity (Fig. 4b). A possible explanation for this could be that F115C and S85C mutant MATR3 exert higher developmental toxicity compared to WT MATR3 in vivo, and thus, any mild protective effect of RRM1 deletion in developmental stages is negated, or perhaps irreversible. However, in adults, both RRM1 and RRM2 deletion significantly extended the lifespan of MATR3 mutant flies (Fig. 4c, d).

We then assessed the effect of RRM1 and RRM2 deletion on total levels of MATR3 WT and mutants. We observed that, especially in the WT background, the total levels of ΔRRM1 and ΔRRM2 were significantly higher compared to full-length MATR3 (Additional file 10: Fig S4), indicating that mitigated toxicities in ΔRRM1 and ΔRRM2-expressing flies is not a consequence of reduced protein expression. Next, we asked if removal of either RRM1 or RRM2 domains has any effect on modulating solubility of the MATR3 protein. Analysis of insoluble and soluble fractions showed that RRM2 deletion significantly increased MATR3 solubility (Fig. 4e–h). Deletion of the RRM1 domain, however, had a milder impact on increasing MATR3 solubility. Interestingly, while RRM2 deletion decreased the insolubility of both WT and mutant MATR3, the relative decrease in insolubility was higher in the WT background compared to the mutant background (Fig. 4f–h). This correlated with our longevity analyses, where RRM2 deletion in the WT background extended the lifespan of MATR3 flies more than in the mutant background (Figs. 3e, 4c, d). Overall, our results highlight an imperative role of the MATR3 RNA-binding domains, particularly the RRM2 domain, in mediating MATR3 WT and mutant toxicity.

### Rump, a homolog of hnRNPM, is a strong modifier of MATR3 toxicity in vivo

**Fig. 5:**
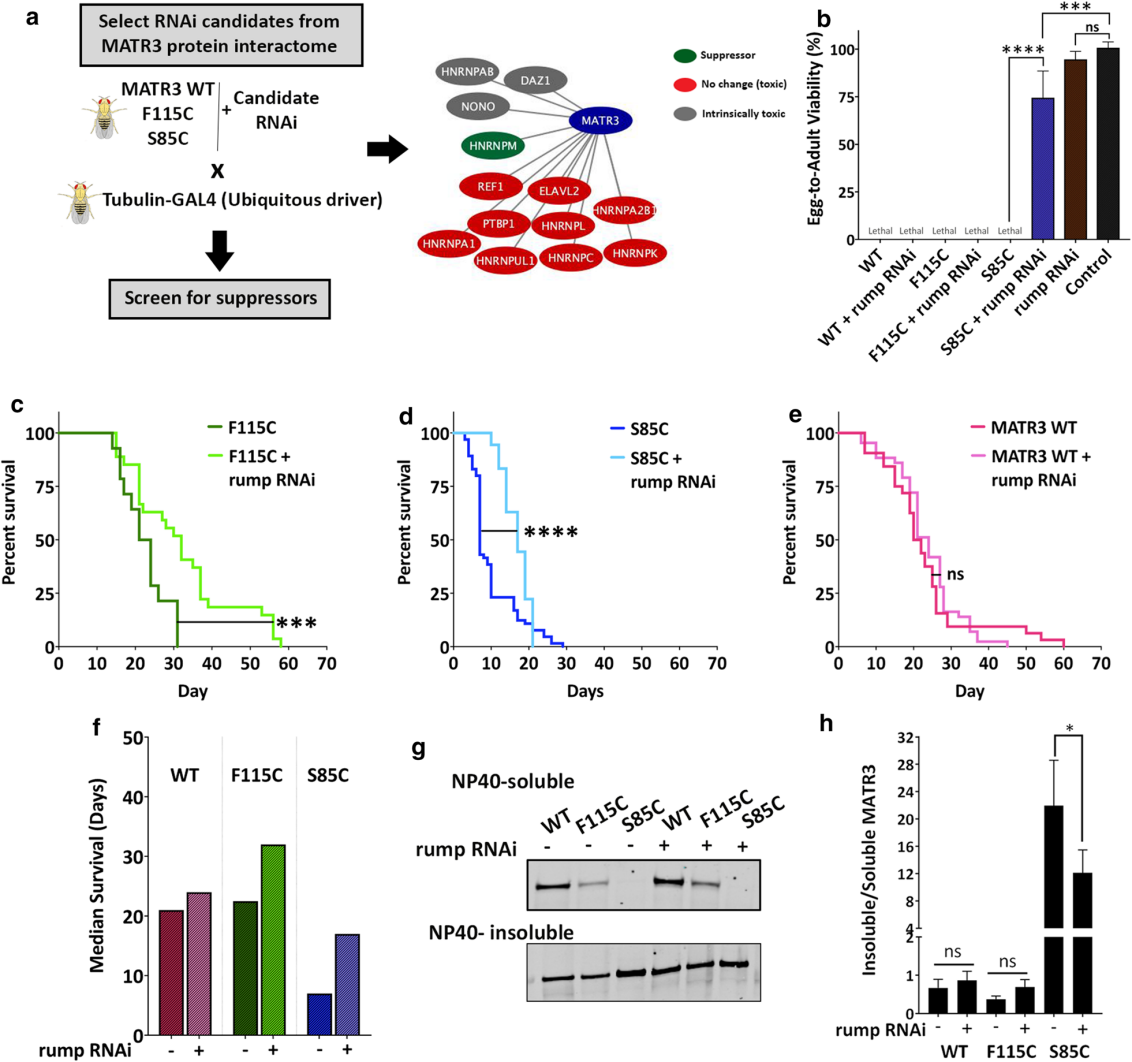
Rumpelstiltskin, homolog of human hnRNPM, is a strong modifier of MATR3 toxicity. **a** Schematic of candidate RNAi screen in MATR3 *Drosophila* model. Diagrammatic representation of MATR3 protein interactors that were screened for suppression of MATR3-mediated toxicity. hnRNPM homolog in *Drosophila*, Rumpelstiltskin (rump), is a unique suppressor of MATR3 toxicity. **b** Quantification of egg-to-adult viability in flies expressing MATR3 with and without rump RNAi. Constitutive ubiquitous expression of S85C with rump knockdown rescues S85C-mediated developmental toxicity (n = 3, *One*-*way ANOVA*). **c** Kaplan–Meier survival curve of adults ubiquitously expressing MATR3 S85C, **d** MATR3 F115C and **e** MATR3 WT, with and without rump knockdown. Knocking down rump significantly improved longevity of F115C and S85C-expressing flies and had no obvious effect on WT longevity (n = 50, *Log*-*rank Mantel*-*Cox* test). **f** Median survival of MATR3-expressing flies with and without rump KD showed increased median survival upon rump KD in F115C- and S85C-expressing flies **g** Immunoblot of NP40-soluble and NP40-insoluble fractions of MATR3 in *Drosophila* thorax expressing MATR3, with or without rump RNAi, in the muscles. α-tubulin is used as loading control. **h** Quantification of the insoluble/soluble MATR3 protein fractions. Rump KD decreased insolubility of MATR3 S85C (n = 6 per group; *One*-*way ANOVA*). Error bars indicate S.E.M. ***p < 0.001; ****p < 0.0001

To identify modifiers of MATR3 toxicity, we focused on proteins that have been identified as high-confidence interactors of MATR3 in two or more high-throughput studies [26, 36, 37]. Most protein–protein interactors were RNA-binding proteins involved in multiple aspects of RNA metabolism, primarily splicing. We screened the interactome for proteins previously implicated in neurodegenerative diseases, including hnRNP family proteins. We obtained publicly available RNAi lines for the selected candidate genes and screened for those that do not cause any intrinsic toxicity with ubiquitous expression (Additional file 11: Table S1). To perform the screen, we combined MATR3 WT and mutants with each candidate RNAi line, crossed them with the ubiquitous driver and looked for viable adults in the progeny (Fig. 5a).

We identified Rumpelstiltskin (*rump*), the *Drosophila* homolog of human *HNRNPM*, as a strong modifier of MATR3 toxicity (Fig. 5a, b) (Additional file 11: Table S1). hnRNPM is an RNA-binding protein that binds to pre-mRNA and splicing regulator complexes to regulate alternative splicing [38, 39]. Furthermore, hnRNPM is one of the strongest interactors of MATR3, identified through multiple high-throughput protein–protein interaction studies [26, 36, 37, 40]. We observed that knocking down *rump* (Additional file 12: Fig S5A, B) significantly rescued S85C toxicity, as evident from rescue of egg-to-adult viability in S85C flies also expressing rump RNAi (Fig. 5b). Interestingly, among all hnRNPs tested, only rump suppressed the MATR3 toxicity, suggesting a potentially prominent role for hnRNPM in mediating MATR3 toxicity in vivo (Additional file 11: Table S1). To validate this interaction, we knocked down *rump* in adults conditionally expressing MATR3 and assessed longevity. rump knockdown (KD) significantly increased the lifespans of flies expressing F115C- or S85C-mutant MATR3 (Fig. 5c, d, f). rump KD on its own did not exert intrinsic toxicity during development (Fig. 5b), however it did promote decreased longevity in adults compared to controls (Additional file 12: Fig S5C). Interestingly, rump KD did not have any obvious effect on WT lifespan (Fig. 5e, f), indicating a role for hnRNPM in mediating toxicity caused by pathogenic mutations in MATR3. This evidence further highlights the disease relevance of a MATR3 and hnRNPM genetic interaction in vivo. Furthermore, rump KD decreased the insolubility of MATR3 S85C in muscles (Fig. 5g, h), suggesting a potential mechanism for alleviating MATR3 toxicity in vivo.

### hnRNPM genetically and physically interacts with MATR3 via its RRM2 domain in mammalian cells

**Fig. 6:**
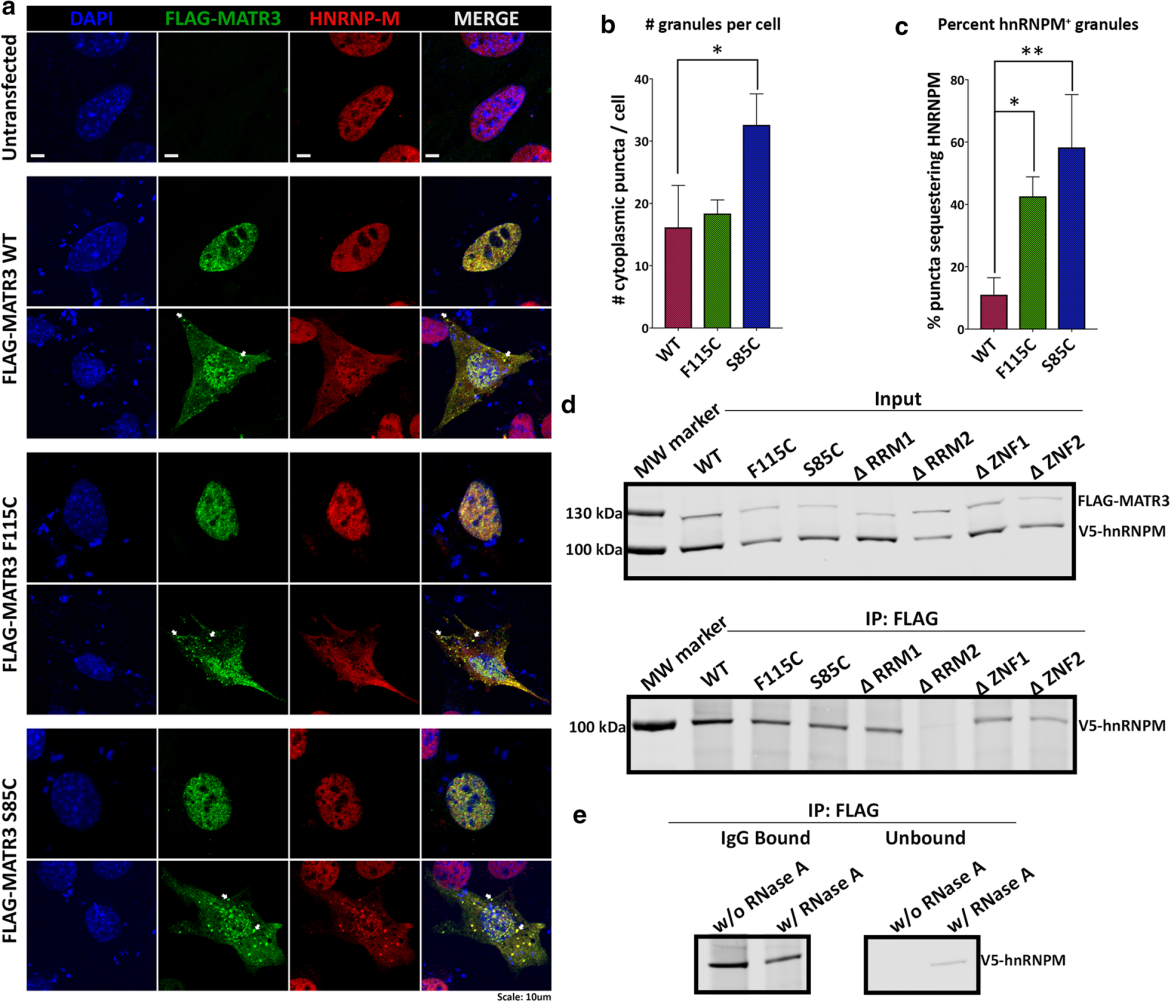
hnRNPM physically interacts with MATR3 in mammalian cells. **a** C2C12 cells ectopically expressing WT and mutant FLAG-MATR3 showed colocalization of FLAG-MATR3 (green) and endogenous hnRNPM (red) in the nucleus and cytoplasm. White arrows point to sequestration of endogenous hnRNPM with FLAG-MATR3 in cytoplasmic granules. **b** Quantification of number of cytoplasmic granules per cell showed that cell expressing F115C and S85C mutant MATR3 had increased formation of cytoplasmic granules (n = 6 per group; *One*-*way ANOVA*). **c** Quantification of percentage cytoplasmic granules that are positive for hnRNPM. Cells ectopically expressing mutant MATR3 showed increased sequestration of hnRNPM in cytoplasmic granules compared to WT (n = 6 per group; *One*-*way ANOVA*). **d** Immunoblot of co-immunoprecipitation of FLAG-MATR3 from HEK293T cells expressing FLAG-MATR3 and V5-hnRNPM. Immunoprecipitation with FLAG antibody showed V5-hnRNPM pulled down with FLAG-MATR3 WT, F115C and S85C. RRM2 deletion disrupts interaction between FLAG-MATR3 and V5-hnRNPM. **e** Immunoblot of lysates immunoprecipitated with FLAG antibody and treated with RNase A. Treatment with RNase A decreases interaction between FLAG-MATR3 WT and V5-hnRNPM (left). Elution of V5-hnRNPM in the unbound fraction after RNaseA treatment (right). Error bars indicate S.E.M. *p < 0.05; **p < 0.01

To identify functional interactions between MATR3 and hnRNPM, we turned to mammalian cell systems. Mouse myoblast C2C12 cells are particularly susceptible to MATR3 overexpression, as ectopic expression of MATR3 results in cytoplasmic mislocalization of MATR3 and accumulation into cytoplasmic granules in a subset of cells (Fig. 6a, b). We also observed that overexpression of the S85C mutation led to significantly increased accumulation of MATR3 into cytoplasmic granules compared to WT overexpression (Fig. 6b). Moreover, we observed clear colocalization between MATR3 and endogenous hnRNPM in the cytoplasmic granules, suggesting that MATR3 overexpression concurrently leads to mislocalization of hnRNPM and sequestration into cytoplasmic granules (Fig. 6a). Interestingly, overexpression of F115C- and S85C-mutant MATR3 caused a higher degree of sequestration of hnRNPM into the cytoplasmic granules compared to WT (Fig. 6c). This correlates with selective suppression of MATR3-mutant toxicity by hnRNPM KD in our *Drosophila* models, supporting the hypothesis that hnRNPM is important for suppressing MATR3 toxicity in a mutation-dependent manner.

While hnRNPM was shown to interact with wildtype MATR3 through mass spectrometric studies [26, 36, 37], we sought to assess the physical interaction between hnRNPM and mutant MATR3. Co-immunoprecipitation revealed a physical interaction between hnRNPM and WT MATR3 as well as F115C and S85C mutants (Fig. 6d). Interestingly, deletion of the RRM2 domain interrupted this interaction, suggesting that the RRM2 domain is required for mediating interaction between MATR3 and hnRNPM (Fig. 6d). To investigate if this interaction is RNA-dependent, we treated the immunoprecipitated lysate with RNaseA to degrade the RNA. Treatment with RNaseA decreased the interaction between MATR3 and hnRNPM in mammalian cells (Fig. 6e), suggesting that these proteins interact, at least partially, through binding to shared RNA targets.

### MATR3 and hnRNPM share common transcriptomic targets

**Fig. 7:**
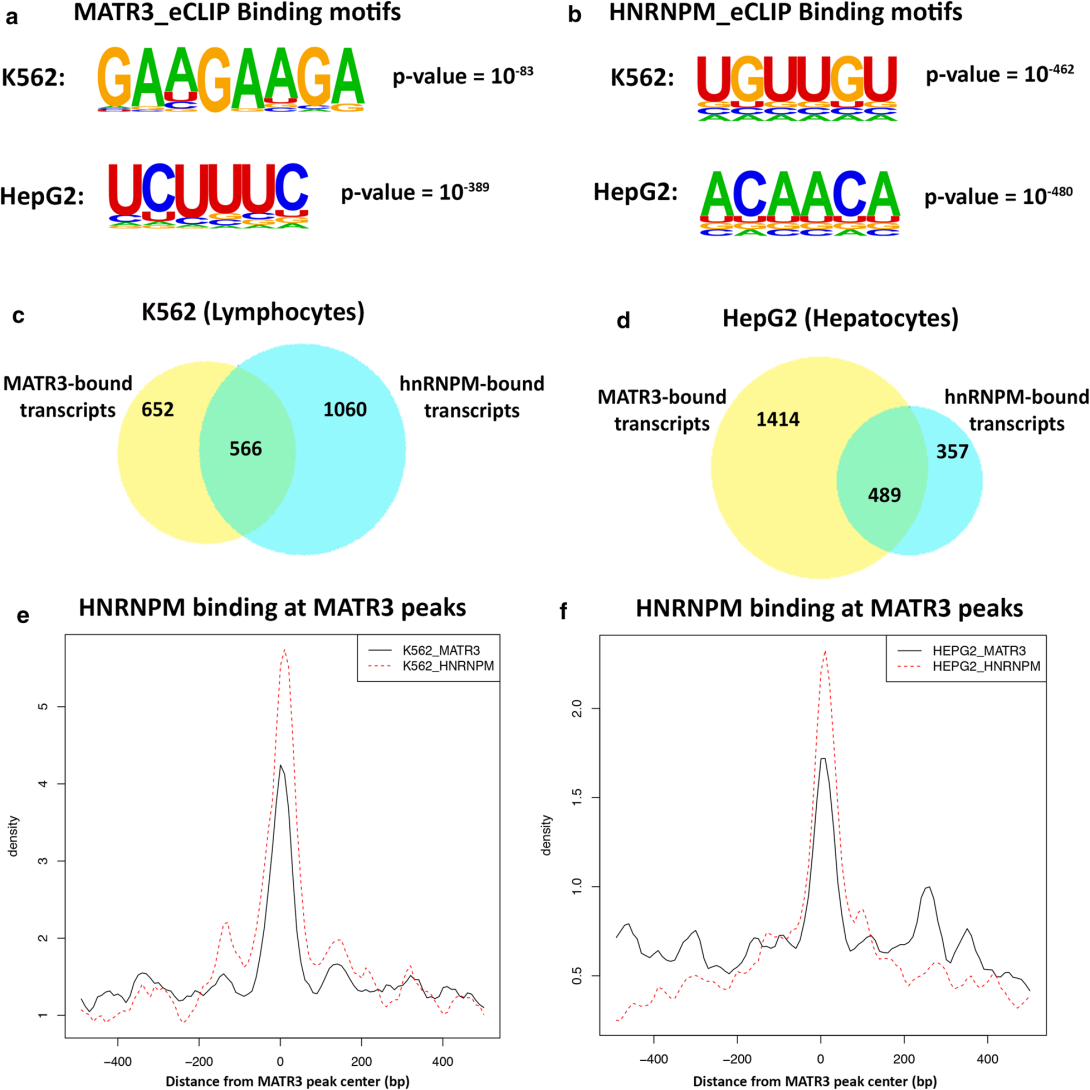
MATR3 and hnRNPM bind to shared transcriptomic targets. **a**MATR3 binding sites are significantly enriched primarily in AAGAA and UCUUU motifs in both K562 and HepG2 cells. MATR3 eCLIP (ENCODE) from K562 cells shows highest enrichment of AAGAA motif with p-value = 10^−83^, and MATR3 eCLIP from HepG2 cells shows highest enrichment of UCUUC motif with p-value = 10^−389^. **b** hnRNPM binding sites (ENCODE) are significantly enriched primarily in UGUUG and ACAAC motifs in both K562 and HepG2 cells. hnRNPM eCLIP from K562 cells shows highest enrichment of UGUUG motif with p-value = 10^−462^, and hnRNPM eCLIP from HepG2 cells shows highest enrichment of ACAAC motif with p-value = 10^−480^. **c** Venn diagram representing transcriptomic targets from in silico analysis of MATR3 eCLIP and hnRNPM eCLIP in K562 cells (lymphocytes) and **d** HepG2 cells (hepatocytes). Significantly enriched MATR3 and hnRNPM eCLIP peaks were identified (p-value < 0.05, fold change ≥ 4) and annotated to corresponding transcript. MATR3 and hnRNPM share transcriptomic targets in both cell types. **e**, **f** Read density plots showing normalized read density of hnRNPM eCLIP centered at significantly enriched MATR3 eCLIP peaks in **e** K562 cells and in **f** HepG2 cells. Read densities were normalized within a ± 400 nucleotide (nt) window around MATR3 peak center

We hypothesized that MATR3 and hnRNPM functionally interact to regulate metabolism of shared RNA targets and that dysregulation of these transcripts possibly leads to disease pathogenesis. We employed an in silico approach to further elucidate this functional interaction. We compared published eCLIP datasets [34] for MATR3 and hnRNPM from two different cell types, K562 (lymphocytes) and HepG2 (hepatocytes), and mined transcripts bound by both proteins (Additional file 4: Tables S2, Additional file 5: Tables S3). Motif discovery across significantly enriched eCLIPs peaks for MATR3 and hnRNPM revealed unique but distinct motifs for MATR3 and hnRNPM. MATR3 binding sites were enriched in AGAAG and UCUUC motifs (Fig. 7a), while hnRNPM binding sites were enriched in UGUUG and ACAAC motifs (Fig. 7b), indicating that these proteins bind to unique motifs within their respective transcript targets. Importantly, we found that MATR3 and hnRNPM share appreciable overlap in the transcripts that they bind (Fig. 7c, d) (Additional file 9: Tables S3). In K562 cells ~ 46% of MATR3-bound transcripts are also bound by hnRNPM (Fig. 7c); in HepG2 cells, ~ 26% of MATR3-bound transcripts are also bound by hnRNPM (Fig. 7d). We then compared the binding patterns of MATR3 and hnRNPM using their read density across the commonly shared transcripts. Considering a window of 300 nucleotides around significantly enriched (*p* < 0.05 and fold-change ≥ 4) MATR3 peaks from K562 and HepG2 cells, we observed that hnRNPM read density is highly enriched around MATR3 peak centers in both cell types (Fig. 7e, d). The vice versa was also observed, in that, MATR3 read densities were highly enriched around hnRNPM peak centers (Additional file 13: Fig S6A, B), suggesting that MATR3 and hnRNPM bind in close proximity to each other on the shared transcript.

**Fig. 8:**
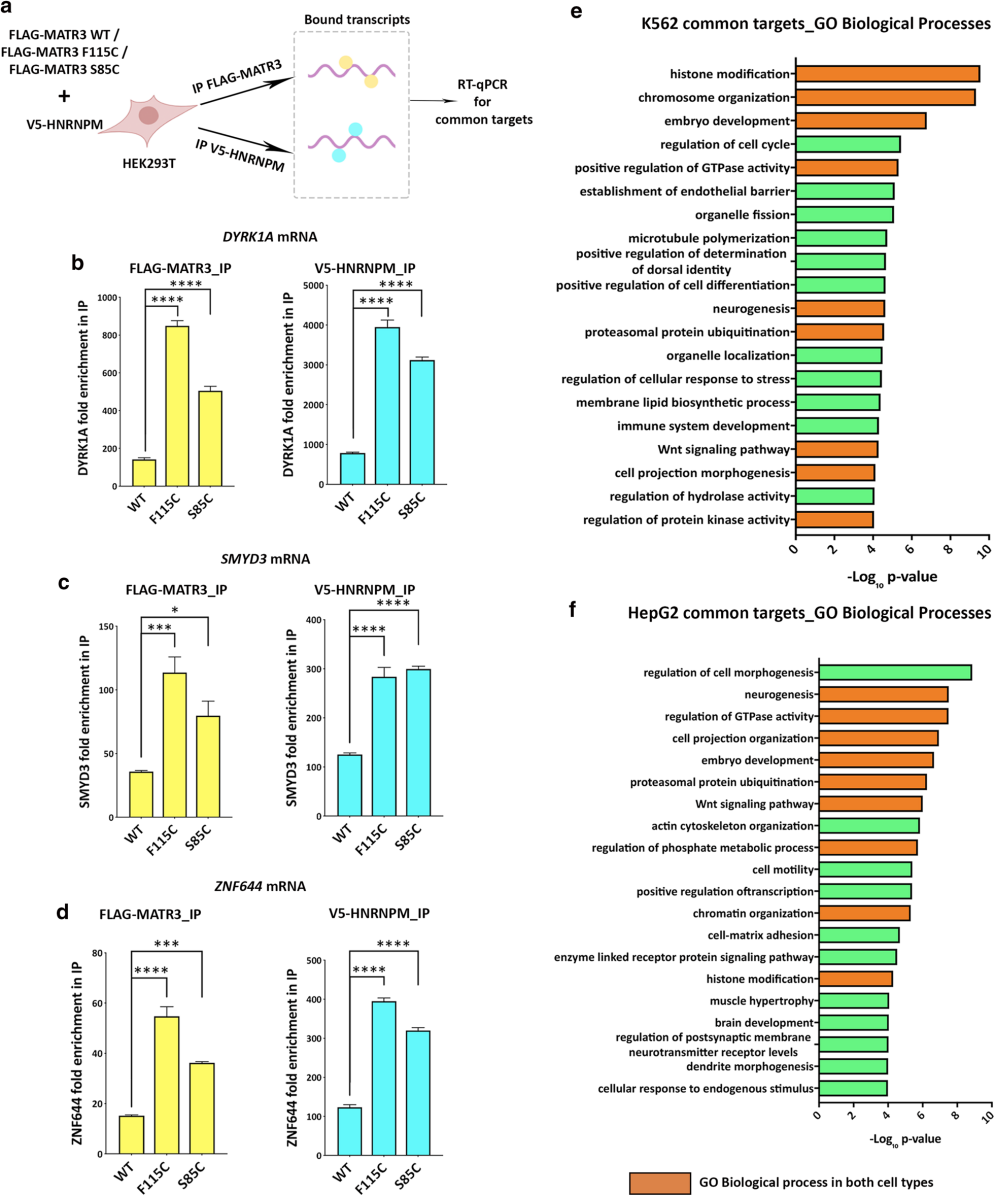
ALS-causing mutations in MATR3 cause aberrant RNA-binding of MATR3 and hnRNPM to transcriptomic transcripts. **a** Schematic of RNA-immunoprecipitation to probe for physical interaction between MATR3 WT, F115C and S85C and hnRNPM. **b–d** Fold change differences in RNA targets immunoprecipitated with FLAG-MATR3 and V5-HNRNPM from cells expressing MATR3 WT and ALS-causing mutations F115C and S85C (n = 3-4 per group; *One*-*way ANOVA*). (**b, left**) *DYRK1A*, (**c, left**) *SMYD3,* and (**d, left**) *ZNF644* mRNA show significantly higher enrichment when immunoprecipitated with F115C and S85C mutants compared to MATR3 WT. (**B, right**) *DYRK1A*, (**C, right**) *SMYD3*, and (**D, right**) *ZNF644* mRNA also show significantly higher enrichment when immunoprecipitated with hnRNPM in cells expressing F115C and S85C groups compared to MATR3 WT. **e** Top 20 unique GO:Biological Process terms that are enriched in gene ontology assessment of shared transcriptomic targets from K562 cells and **f** HepG2 cells. Green bars indicate biological processes unique to the cell type. Orange bars indicate biological processes commonly enriched in both cell types. Error bars indicate S.E.M. *p < 0.05; **p < 0.01; ***p < 0.001; ****p < 0.0001

To further investigate the nature of MATR3 and hnRNPM interaction with shared transcripts, specifically in the context of ALS/myopathy-linked mutations in MATR3, we performed RNA-immunoprecipitation of MATR3 and hnRNPM in HEK293T cells expressing either MATR3-WT or disease-linked mutations MATR3-F115C and MATR3-S85C (Fig. 8a). RT-qPCR for rationally-selected targets from the shared transcriptome, particularly those that show high binding affinity to both MATR3 and hnRNPM, and additionally, have been previously associated to neurodegeneration in ALS including *DYRK1A*, *SMYD3* and *ZNF644*, revealed that F115C and S85C mutants exhibit significantly higher binding to these transcripts compared to WT (Fig. 8b–d). Importantly, we found that hnRNPM also exhibited significantly higher binding to the same transcripts in cells expressing F115C and S85C compared to cells expressing MATR3-WT (Fig. 8b–d). This suggests that disease-causing mutations in MATR3 lead to aberrant RNA-binding to MATR3 and, concurrently, to its interacting-partner, hnRNPM. Gene ontology analysis of all common targets (Additional file 6: Tables S4) revealed that the top 20 most enriched unique biological processes shared between the two cell types included significant processes such as neurogenesis, proteasomal protein ubiquitination, histone modification & chromosome organization (Fig. 8h, i). Furthermore, gene ontology analysis on the basis of disease associations indicated that the shared targets are enriched in genes associated with neurodevelopmental disorders (Additional file 6: Tables S4). These results indicate that MATR3 and hnRNPM both bind to, and thus may co-regulate, transcripts that have important functions in nervous system development and maintaining cellular/neuronal health. Thus, it is likely that aberrant regulation of these transcripts, and consequently these processes, caused by mutations in MATR3 result in disease pathogenesis.

We sought to further validate this hypothesis in our in vivo model. We rationally-selected candidate targets from the MATR3-hnRNPM shared transcriptome, focusing on candidates that have previously been shown by other studies to be regulated by MATR3 in mammalian cells [20, 27, 34], and additionally exhibit disease-relevance in ALS and other neurodegenerative disorders. Assessment of levels of candidate transcripts, including Dystrophin (*Dys*), Ataxin-1 (*Atx*-*1*), Adenylate kinase 3 (*Adk3*) and Sialyltransferase (*SiaT*), revealed significantly increased mRNA levels in mutant MATR3-expressing flies compared to control (Additional file 14: Fig S7), while it remained unchanged in the WT compared to control. Furthermore, RNA-immunoprecipitation of MATR3 and hnRNPM in human cells expressing either MATR3-WT or F115C and S85C mutations, showed significantly higher binding of *UTRN* mRNA (*Dys* homolog) to both F115C and S85C mutants compared to WT (Additional file 15: Fig S8). Interestingly, *UTRN* mRNA also showed significantly higher binding to hnRNPM in cells expressing either F115C or S85C mutations compared to cells expressing MATR3-WT (Additional file 15: Fig S8). These results further support our idea that hnRNPM modulates mutant MATR3 toxicity, and both proteins genetically and physically interact to regulate common targets.

## Discussion

We comprehensively characterized MATR3-mediated toxicity in a transgenic *Drosophila* model. *Drosophila* is a versatile model system that has been used for over a century to study the molecular mechanisms of key biological functions given their genetic and overall experimental tractability [41]. In the context of ALS, *Drosophila* has been extensively used to model both genetic (mutations in ALS-associated genes) and environmental insults (traumatic brain injury) that lead to disease pathogenesis [42–45].

Although similar in structure and function to *Drosophila* orthologs of many RNA-binding proteins, there is no known *Drosophila* ortholog for MATR3. Thus, our transgenic model allowed us to investigate expression of wild type and mutant MATR3 in vivo without interference from endogenous protein. Ubiquitous expression of MATR3 in *Drosophila* negatively impacted viability during development and in adults, supported by underlying neuromuscular junction defects and motor dysfunction. Tissue-specific expression revealed higher susceptibility to MATR3 expression in muscles and motor neurons. Particularly in muscles, MATR3 expression led to early development of motor deficits that persisted with aging and eventually decreased longevity. This age-dependent decline in motor function was also reported in a transgenic mouse model expressing the F115C mutant [31]. Another striking similarity between the two models is development of myopathic histopathological changes underlying the motor deficits. In *Drosophila*, expression of MATR3 in muscles results in atrophy of the indirect flight muscles, exacerbated by the F115C and S85C mutations.

A recurring theme in the behavioral assessment of our transgenic MATR3 model is that expression of the wild type protein has comparable toxicity to the mutants. One possible explanation could be that since *Drosophila* lack any known endogenous ortholog of MATR3, we are observing toxic effects of overexpression of a foreign protein. Nevertheless, observations from our deletion mutant models and identification of genetic modifier indicates that MATR3 is an active participant in the genetic/proteomic network that regulates biological processes in *Drosophila* tissues. Thus, while lack of any endogenous MATR3 in flies allowed us to comprehensively understand the underpinnings of MATR3 biology that drive toxicity in an in vivo model. The toxic effects of MATR3 overexpression are supported by evidence from rat cortical neurons where ectopic expression of either wild type or ALS-associated mutant MATR3 reduces neuron survival [29]. Similarly, in mice, transgenic expression of either wild type or F115C mutant MATR3 results in myopathic changes in pathology, and F115C expression leads to more severe paralysis [31]. Mechanistically, overexpression of wild type and mutant MATR3 impedes nuclear mRNA export in NSC-34 cells, suggesting that MATR3 function is similarly impacted in either condition. Overall, our model and others suggest a potential gain-of-function in MATR3 caused by pathogenic mutations. While MATR3 immunoreactivity appears to be higher in a few ALS and myopathy patient tissues [10, 15], further analysis of patient tissues, and/or discovery of a duplication mutation in MATR3 resulting in similar phenotypes, is required to validate the gain-of-function models.

The sub-cellular localization of MATR3 is unaffected by the mutations in our model. However, the biochemical properties of MATR3 are significantly changed by the mutations. MATR3 protein is typically distributed between detergent-soluble and insoluble fractions. In muscles, the S85C mutation drastically decreases the protein solubility. Our data also alludes to an age-dependent concomitant increase in toxicity and insolubility of MATR3 WT and mutants. However, it is intriguing that the higher insolubility of MATR3-S85C does not correlate with higher overall toxicity compared to WT and F115C suggest there might be additional factors responsible for conferring toxicity. Multiple disease-associated proteins can misfold into toxic conformations that render them insoluble [5, 46, 47]; our finding suggests that the S85C mutation cause toxicity through similar mechanisms. In addition to solubility, assessment of protein stability in vivo demonstrates a higher half-life, particularly of insoluble mutant MATR3 compared to wild type. This suggests that MATR3 mutations result in more stable and insoluble species that might be disrupting protein function. Interestingly, both mutations result in cysteine residues. Cysteine residues are capable of forming stable disulfide bridges, which might explain the increased stability and insolubility of MATR3 mutants compared to wild type. TDP-43, another protein that forms detergent-insoluble species [48], can form more insoluble species in response to oxidative stress. This occurs because of oxidization of cysteine residues, which results in crosslinking of TDP-43 through disulfide bridges [49]. Comprehensive characterization of the biochemical and biophysical properties of wild type and mutant MATR3, particularly in response to stress, may help elucidate disease pathogenesis.

Since none of the pathogenic mutations in MATR3 lie within the known functional domains of the protein, the mutations could be acting in *cis* with the functional domains to negatively impact MATR3 function. By generating and characterizing transgenic lines with deletion mutations in each functional domain of the protein, we showed that MATR3 toxicity in vivo is governed by its RNA-binding domains. Indeed, RRM1 and RRM2 deletion strongly mitigated MATR3 toxicity, which was protective not only in the wild type background, but also to some extent in the mutant background. While both RRM1 and RRM2 domains can mediate regulation of splicing and maintenance of mRNA stability, RNA-binding ability itself has only been shown for the RRM2 domain [23]. Previous studies have shown that RNA-binding ability is essential for causing the toxicities associated with FUS [50, 51] and TDP-43 [52] and mutating the RRM domain abolishes the toxic effects. While RRM1/2 deletion exerts a protective effect on both WT and mutant toxicity, it is intriguing that RRM1/2 deletion does not rescue toxicity to the same extent in the mutants compared to WT. This suggests that additional functional domains of MATR3 protein might contribute to mutant MATR3 toxicity. While deletion of either RRM1 or RRM2 domain partially alleviates mutant MATR3 toxicity individually, it remains to be seen if both RRM1 and RRM2 domains act synergistically to exert toxicity. Simultaneously, mutations in MATR3 might induce structural changes including toxic protein misfolding that promote toxicity that is partially, but not completely, alleviated by deletion of the RRM domains. It is especially curious that both F115C and S85C mutations lie in the N-terminal of the protein that is currently still uncharacterized with respect to its function and molecular interactions. Thus, we cannot rule out the possibility that RRM-independent mechanisms also contribute to mutant MATR3 toxicity. Future investigations that assess MATR3-mediated toxicity when RNA-binding is inhibited by point mutations in the RRM domains, as well as when both RRM1 and RRM2 domains are inactivated, will shed further light on the role of these functional domains and importance of RNA-binding in mutant MATR3.

A previous study in rat cortical neurons did not observe any change in toxicity exerted by MATR3 when the RRM domains are deleted [29]. The difference could be attributed to the different model systems used—cultured rat cortical neurons versus a whole-animal *Drosophila* model system. A main difference between our models is the absence of endogenous MATR3 in *Drosophila*, whereas, rat cortical neurons do have an endogenous MATR3. Thus, it is likely that presence of endogenous MATR3 is sufficient to mask the effect of RRM deletion in rat cortical neurons. Contrastingly, in flies, overexpression of MATR3 WT and mutants, in the absence of interference from endogenous protein, leads to aberrant binding and dysregulation of RNA which is inhibited by removing RNA-binding ability. Additionally, MATR3 toxicity was exacerbated by deletion of either zinc-finger domain (ZF1 and ZF2) in our model. The functions of these domains in MATR3 are less understood compared to the RRM domains. Both zinc-finger domains are required for mediating MATR3 interaction with DNA [53]. Thus, one possibility is that the zinc-finger domains interact with DNA and regulate transcription and chromosomal arrangements that are imperative for viability in our model. Another possible explanation could be that deletion of the zinc-finger domains re-distributes the protein and enhances its RNA-binding affinity, and concurrently its RNA-binding toxicity. In fact, it has been shown that removal of zinc-finger (ZF1) domain increased the splicing repressor activity of MATR3 [20]. Thus, deletion of zinc-finger domains could be enhancing the toxic gain-of function activity of MATR3, mediated by its RNA-binding and RNA dysregulation.

RNA-binding proteins form dynamic and complex networks to perform their functions in an age- and tissue-dependent manner [54]. The multisystem disease etiology of MATR3 strongly indicates that there are other gene/protein modifiers that act alongside MATR3 in mediating disease pathogenesis. One of the strengths of the *Drosophila* model is the ability to perform screens to identify genetic interactors. Unsurprisingly, the MATR3 protein interactome reveals interactions with other RNA-binding proteins involved primarily in RNA splicing [26, 36, 37]. Through our candidate screen to elucidate genetic interactions between MATR3 and its interactome, we discovered *Drosophila* Rumpelstiltskin (rump), the homolog of human hnRNPM, as a strong modifier of MATR3 toxicity. Interestingly, our work is not the first to discover a potential role for rump in ALS. Investigation of hnRNPs that modify TDP-43 toxicity in a *Drosophila* model also identified rump as a strong modifier [55], suggesting that MATR3 and TDP-43 could have converging pathological mechanisms. Mass spectrometry-based studies have consistently found hnRNPM to be one of the top interactors of MATR3 [26, 36, 37]. A study comparing the MATR3 protein interactome in deletion mutants found that interaction with hnRNPM is diminished when the MATR3 RRM2 domain is deleted [36]. Consistent with this finding, using co-immunoprecipitation we showed that wild type and mutant MATR3 interacts with hnRNPM, and that this interaction is mediated by the RRM2 domain.

hnRNPM is a splicing factor that associates with pre-spliceosome and mature spliceosome complexes to regulate alternative splicing [38, 39]. A recent study showed that MATR3 and hnRNPM interact in a large assembly of splicing regulators (LASR) complex in conjunction with the brain-specific RBFOX splicing protein [40]. Accordingly, we showed that MATR3 and hnRNPM interaction in vitro occurs in an RNA-dependent manner. Furthermore, we discovered that there is a significant overlap in transcripts co-bound by these proteins, and importantly, we showed that mutations in MATR3 lead to aberrant RNA-binding of candidate transcripts to both MATR3 and hnRNPM. Aberrant RNA-binding might lead to dysregulation in the processing of these transcripts, and consequently the biological processes regulated by them. Interestingly, the targets we found to have enhanced interaction with both MATR3 and hnRNPM in cells expressing disease-causing mutations in MATR3, including *DYRK1A* and *SMYD3*, are also prime RNA targets of other ALS-associated RBPs, FUS and TDP-43, and additionally, have been associated with RNA dysregulation in ALS [56–61].

Transcripts that are co-bound by MATR3 and hnRNPM converge primarily on developmental processes, particularly those imperative for nervous system development including neurogenesis and neuronal differentiation. Considering the importance of tight regulation of alternative splicing during development, it is not surprising that MATR3 and hnRNPM might co-regulate developmentally important transcripts. It remains to be determined if any of these processes are perturbed in age-related neuromuscular degeneration caused by MATR3 mutations. Following neurodevelopment, chromatin remodeling and histone modification were the most prominent biological processes in our analysis. In post-mitotic neurons, these processes might be actively involved in regulating the cellular response to aging and stress. In fact, TDP-43 was recently implicated in sequestering the chromatin remodeling complex, Chd1, in *Drosophila* and disrupting its function in activating stress response genes [62]. An attractive candidate transcript shared between MATR3 and hnRNPM is HDAC4, an enzyme that modifies chromatin through histone deacetylation (Additional file 5: Table S3). In the SOD1 model of ALS, HDAC4 plays a protective role in the neuromuscular junction and muscle innervation [63]. Other biological processes with strong evidentiary basis in neurodegenerative disease biology include the ubiquitin–proteasome system [64] and Wnt signaling pathway [65, 66]. It could be inferred that these biological processes are perturbed by dysregulation of MATR3-bound transcripts due to the disease-causing mutations. Future studies focusing on using CLIP-seq approaches for identifying differences in MATR3 wild type and mutant transcriptomic binding in the context of hnRNPM levels and/or disruption of hnRNPM-binding, particularly in neuronal cells, would be important for deciphering the mechanisms of RNA dysregulation in MATR3-ALS.

## Conclusions

In summary, we propose that our *Drosophila* model of MATR3 is a robust system to elucidate the basis of MATR3 biology and mechanisms underlying MATR3-mediated neuromuscular degeneration. We show that expression of MATR3 is toxic in *Drosophila*, with muscles and motor neurons exhibiting selective vulnerability to MATR3 expression. Importantly, we show that MATR3 wildtype and mutant toxicity is mediated by its RNA-binding domains, particularly the RRM2 domain. Thus, our model strongly suggests that mutations in MATR3 could be causing aberrant regulation of its RNA targets. These findings highlight the importance of further studies investigating the nature of MATR3-RNA interactions under varying disease conditions (mutations) and physiological conditions (stress, aging) to fully understand the role of RNA binding in regulating physiological functions of MATR3. We also show that mutations in MATR3 could be driving toxicity through its interaction with the splicing factor hnRNPM in an RNA-dependent manner. We extrapolate our observations to identify transcriptomic targets that are commonly bound by MATR3 and hnRNPM. We show that disease-causing mutations in MATR3 lead to increased, aberrant RNA-binding of both MATR3 and hnRNPM to shared transcripts, and also show that levels of candidate targets are altered in our *Drosophila* model, demonstrating that our in silico findings are translated in mammalian cells and our in vivo model systems. We propose that mutations in MATR3 could lead to dysregulation of the shared transcriptome and consequently the associated biological processes. Further studies focusing on how mutations in MATR3 alter regulation of these transcriptomic candidates and the role of hnRNPM could help shed light on mechanisms of disease pathogenesis and identify targets for therapeutic intervention.

## Supplementary information

**Additional file 1: Table S5**. List of primers used for pCMVTag2B FLAG-MATR3 variants sequence verification.

**Additional file 2: Table S6**. List of *Drosophila* lines used in this study and their sources.

**Additional file 3: Table S7**. List of primers and probes for real-time quantitative PCR.

**Additional file 4: Table S2**. Annotated list of enriched peaks from MATR3 and hnRNPM eCLIP (ENCODE) in K562 and HepG2 cells.

**Additional file 5: Table S3.** List of transcripts bound by MATR3 and hnRNPM in K562 and HepG2 cells.

**Additional file 6: Table S4**. Top 20 unique GO:Biological Process terms for MATR3 and hnRNPM shared transcriptomic targets in K562 and HepG2 cells.

**Additional file 7: Figure S1.** MATR3 localizes in nucleus in *Drosophila* model. Larval ventral nerve chord (VNC) immunostained for FLAG in larvae expressing FLAG-MATR3 WT and mutants, F115C and S85C, in motor neurons. FLAG-MATR3 WT and mutants localize to the nucleus (Dapi) in VNC cells.

**Additional file 8: Figure S2**. MATR3 expression in *Drosophila* motor neurons and muscles reduces longevity of flies. **(A)** Tabular representation of number of days it takes for 25%, 50% and 75% death in flies expressing MATR3 WT and mutants in motor neurons and **(B)** muscles. At each 25%, 50% and 75% death stages, flies expressing MATR3 in either tissue die earlier than respective driver-alone controls.

**Additional file 9: Figure S3**. MATR3 expression in *Drosophila* eyes does not cause external or internal degeneration. **(A**) External eye phenotypes of flies expressing MATR3 in the eye, driven by GMR-gal4, at day 1 post-eclosion and **(B)** day 30 post-eclosion. Expression of either WT or mutant MATR3 did not cause any external eye degeneration at early or later time points. **(C)** H&E stained photoreceptors in cross-sections of *Drosophila* eyes. Expression of MATR3 did not result in any internal degeneration.

**Additional file 10: Figure S4**. Total MATR3 protein expression levels in flies expressing ΔRRM1 and ΔRRM2 variants. **(A)** Immunoblot showing total MATR3 protein levels in flies ubiquitously expressing full-length MATR3 WT, F115C and S85C and corresponding ΔRRM1 and ΔRRM2 deletion mutations. **(B)** Quantification of replicate western blots shows increased levels on ΔRRM1 and ΔRRM2-MATR3 compared to full-length MATR3. (n = 4 per group; *One*-*way ANOVA*). Error bars indicate S.E.M. *p < 0.05, **p < 0.01, ***p < 0.001, ****p < 0.0001.

**Additional file 11: Table S1**: List of candidate genes and their respective *Drosophila* homologs used for RNAi screen.

**Additional file 12: Figure S5**. Knockdown of *rump* in *Drosophila* reduces adult longevity. **(A)** Immunoblot showing reduced rump protein levels in rump RNAi line. **(B)** Quantification of replicate western blots to confirm reduced rump levels in rump RNAi flies, driven by Tub-Gal4, compared to driver-alone control (n = 3; *Kruskall*-*Wallis test*). **(C)** Kaplan–Meier survival curve of adults ubiquitously expressing rump RNAi. Knockdown of rump conditionally in adults reduced longevity of flies compared to driver-alone control *(*n = 50, *Log*-*rank Mantel*-*Cox test*) Error bars indicate S.E.M. **p < 0.01, ****p < 0.0001.

**Additional file 13: Figure S6.** MATR3 is enriched at hnRNPM eCLIP peaks. **(A,B)** Read density plots showing normalized read density of MATR3 eCLIP centered at significantly enriched hnRNPM eCLIP peaks in **(A)** K562 cells and in **(B)** HepG2 cells. Read densities were normalized to a ± 400 nucleotide (nt) window around hnRNPM peak center.

**Additional file 14: Figure S7**. Levels of candidate targets from MATR3-hnRNPM shared transcriptome altered in *Drosophila* model. Quantitative graph showing fold change difference in mRNA levels of **(A)** Dystrophin, *Dys*, **(B)** Ataxin-1, *Atx*-*1*, **(C)** Adenylate kinase 3, *Adk3*, and **(D)** Sialyltransferase, *SiaT,* in flies ubiquitously expressing WT and mutant MATR3. mRNA levels of candidate targets are significantly higher in both F115C- and S85C-expressing flies (*Dys* and *Adk3*) or only S85C-expressing flies (*Atx*-*1* and *SiaT*) compared to driver-alone control (n = 5 per group; *One*-*way ANOVA*). Error bars indicate S.E.M. **p < 0.01, ***p < 0.001, ****p < 0.0001.

**Additional file 15: Figure S8**. *UTRN* mRNA binding to MATR3 and hnRNPM is significantly enriched in cells expressing ALS-causing mutations in MATR3. Fold change differences in *UTRN* mRNA immunoprecipitated with FLAG-MATR3 and V5-HNRNPM from cells expressing MATR3 WT and ALS-causing mutations F115C and S85C. *UTRN* binding is significantly enriched to MATR3 (left) and hnRNPM (right) in cells expressing F115C and S85C (n = 3-4 per group; *One*-*way ANOVA*). Error bars indicate S.E.M. *p < 0.05, ****p < 0.0001.

## Data Availability

All data generated or analyzed during this study are included in this published article [and its supplementary information files].

## Abbreviations

ALS: Amyotrophic lateral sclerosis
DLM: Dorsal-longitudinal muscle
FTD: Frontotemporal dementia
GO: Gene ontology
H&E: Hematoxylin and eosin
IDR: Intrinsically disordered region
KD: Knockdown
NMJ: Neuromuscular junction
RRM: RNA recognition motif
VCPDM: Vocal cord and pharyngeal distal myopathy
VNC: Ventral nerve cord
ZNF/ZF: Zinc finger
