# Supplementary figures and images for: RNA-recognition motif in Matrin-3 mediates neurodegeneration through interaction with hnRNPM
Source: Acta Neuropathol Commun. 2020 Aug 18;8:138. doi: 10.1186/s40478-020-01021-5 (PMC7437177; doi:10.1186/s40478-020-01021-5)

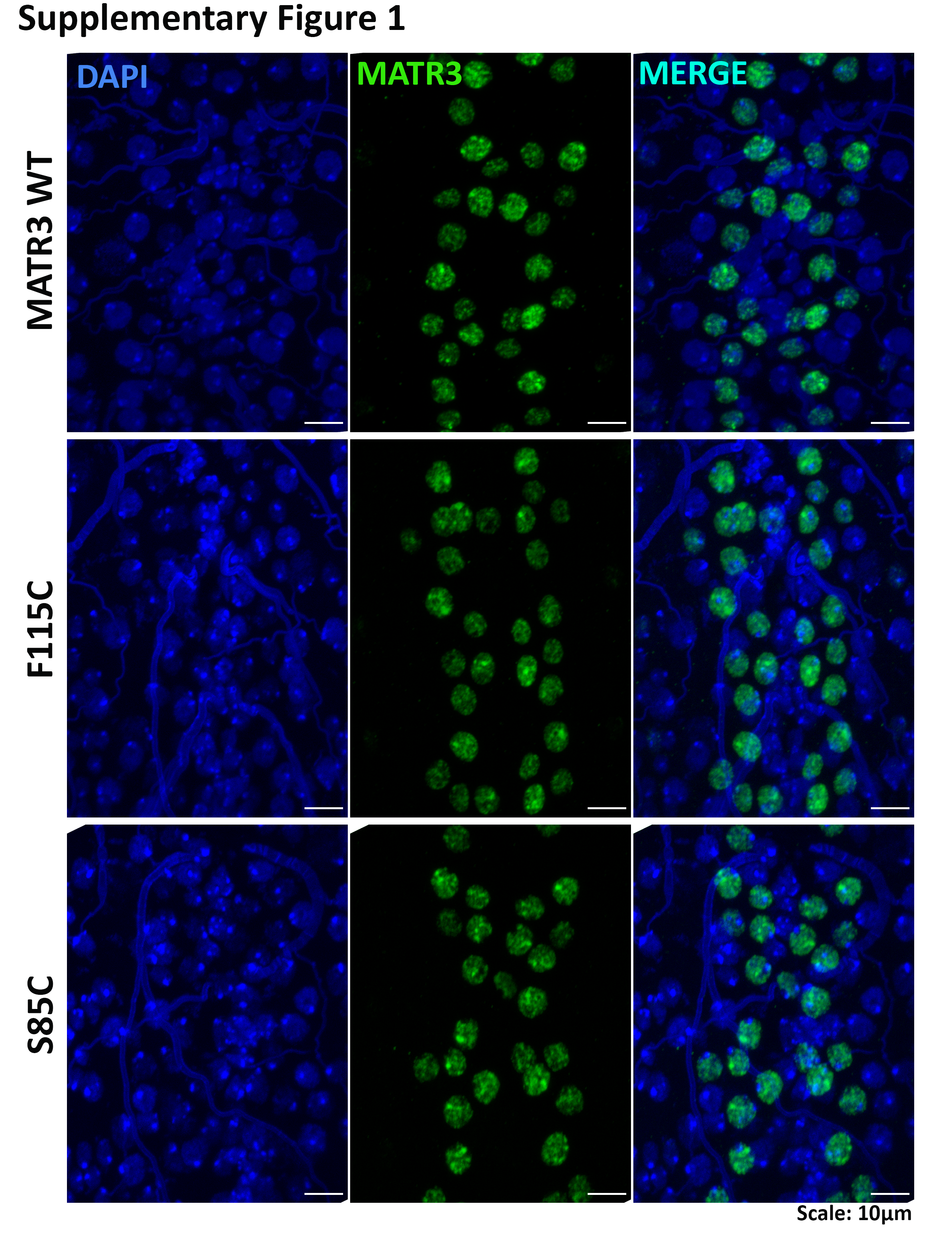

Supplement: Supplementary file 7 — Additional file 7: Figure S1. MATR3 localizes in nucleus in Drosophila model. Larval ventral nerve chord (VNC) immunostained for FLAG in larvae expressing FLAG-MATR3 WT and mutants, F115C and S85C, in motor neurons. FLAG-MATR3 WT and mutants localize to the nucleus (Dapi) in VNC cells. [file 40478_2020_1021_MOESM7_ESM.tif]

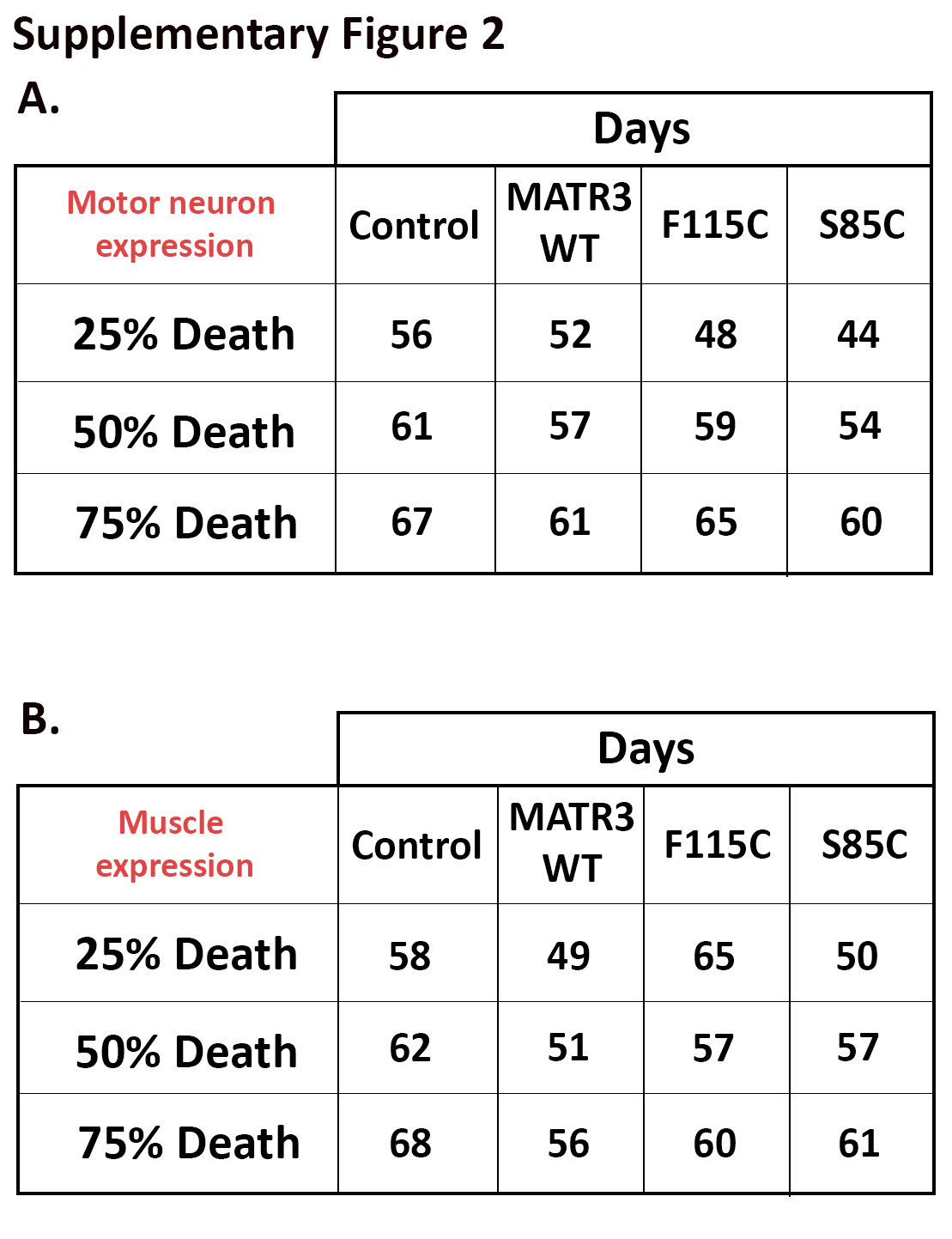

Supplement: Supplementary file 8 — Additional file 8: Figure S2. MATR3 expression in Drosophila motor neurons and muscles reduces longevity of flies. (A) Tabular representation of number of days it takes for 25%, 50% and 75% death in flies expressing MATR3 WT and mutants in motor neurons and (B) muscles. At each 25%, 50% and 75% death stages, flies expressing MATR3 in either tissue die earlier than respective driver-alone controls. [file 40478_2020_1021_MOESM8_ESM.tif]

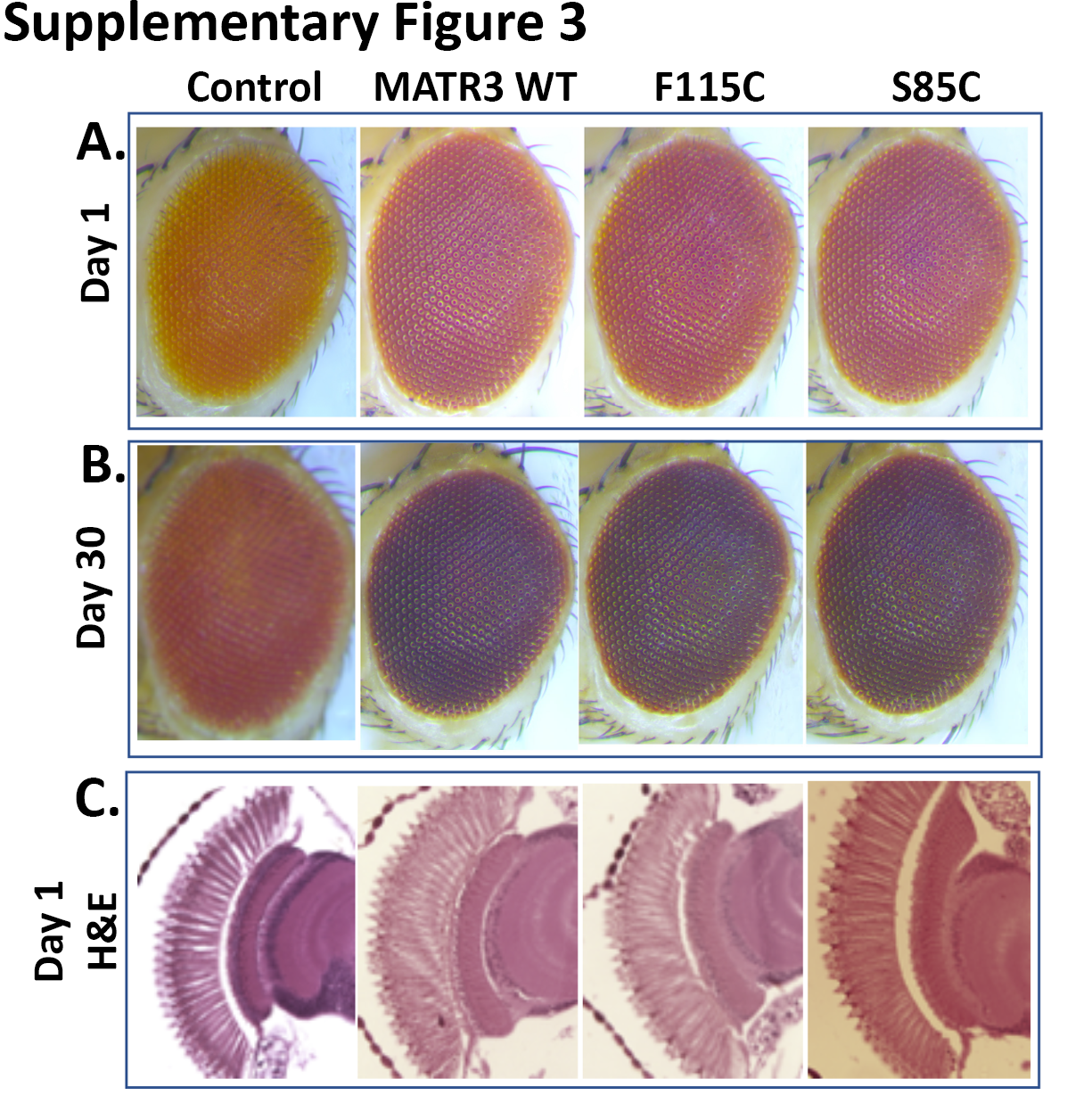

Supplement: Supplementary file 9 — Additional file 9: Figure S3. MATR3 expression in Drosophila eyes does not cause external or internal degeneration. (A) External eye phenotypes of flies expressing MATR3 in the eye, driven by GMR-gal4, at day 1 post-eclosion and (B) day 30 post-eclosion. Expression of either WT or mutant MATR3 did not cause any external eye degeneration at early or later time points. (C) H&E stained photoreceptors in cross-sections of Drosophila eyes. Expression of MATR3 did not result in any internal degeneration. [file 40478_2020_1021_MOESM9_ESM.tif]

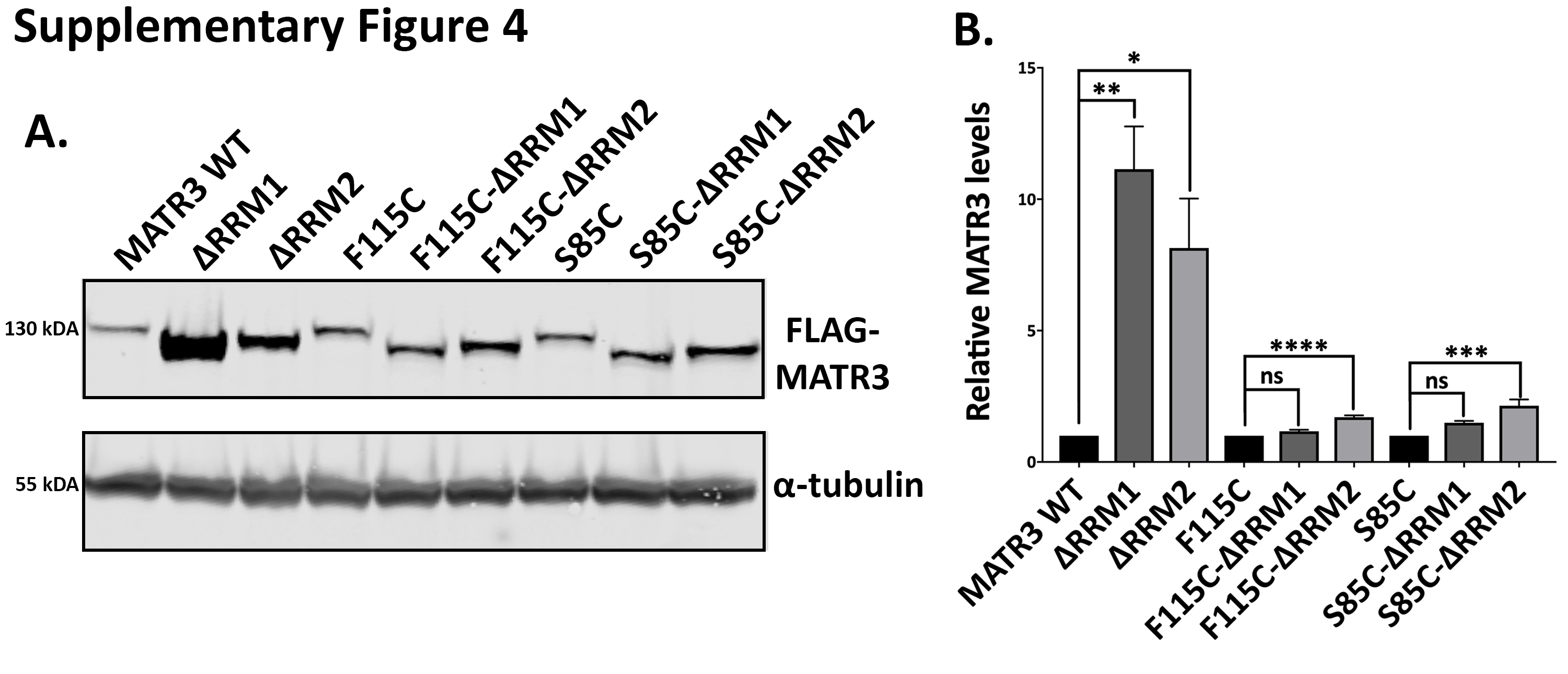

Supplement: Supplementary file 10 — Additional file 10: Figure S4. Total MATR3 protein expression levels in flies expressing ΔRRM1 and ΔRRM2 variants. (A) Immunoblot showing total MATR3 protein levels in flies ubiquitously expressing full-length MATR3 WT, F115C and S85C and corresponding ΔRRM1 and ΔRRM2 deletion mutations. (B) Quantification of replicate western blots shows increased levels on ΔRRM1 and ΔRRM2-MATR3 compared to full-length MATR3. (n = 4 per group; One-way ANOVA). Error bars indicate S.E.M. *p < 0.05, **p < 0.01, ***p < 0.001, ****p < 0.0001. [file 40478_2020_1021_MOESM10_ESM.tif]

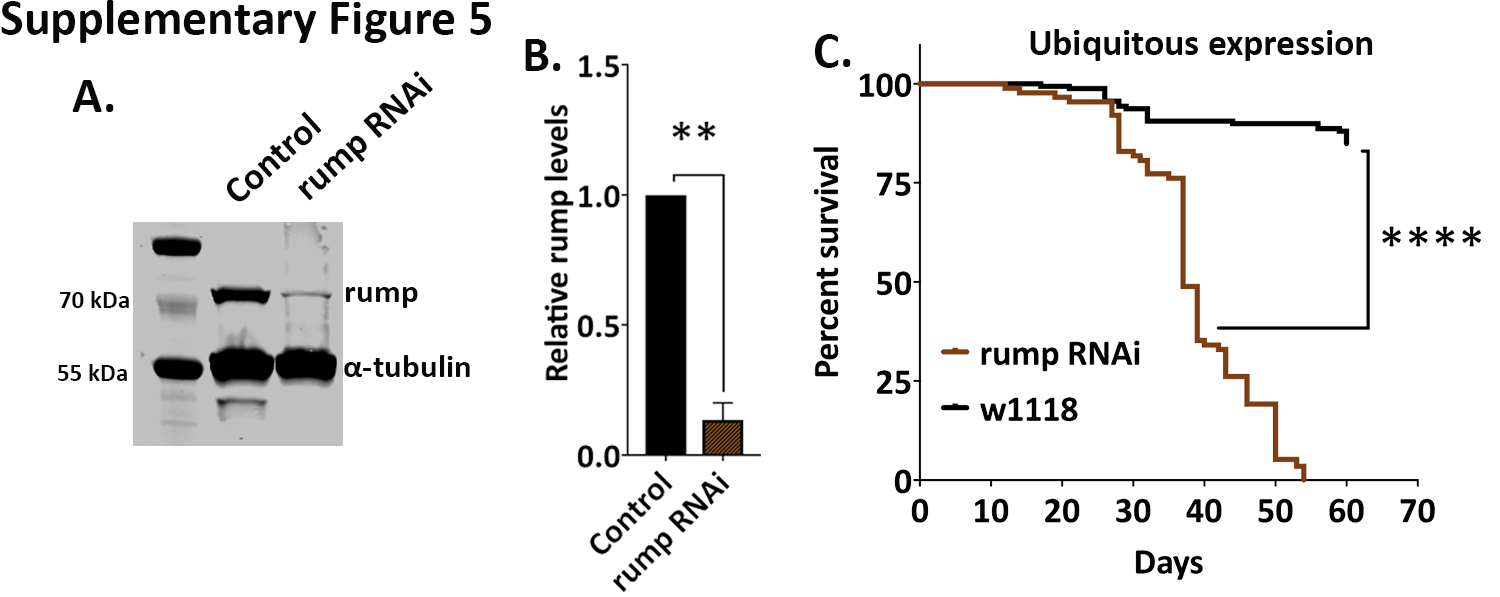

Supplement: Supplementary file 12 — Additional file 12: Figure S5. Knockdown of rump in Drosophila reduces adult longevity. (A) Immunoblot showing reduced rump protein levels in rump RNAi line. (B) Quantification of replicate western blots to confirm reduced rump levels in rump RNAi flies, driven by Tub-Gal4, compared to driver-alone control (n = 3; Kruskall-Wallis test). (C) Kaplan–Meier survival curve of adults ubiquitously expressing rump RNAi. Knockdown of rump conditionally in adults reduced longevity of flies compared to driver-alone control (n = 50, Log-rank Mantel-Cox test) Error bars indicate S.E.M. **p < 0.01, ****p < 0.0001. [file 40478_2020_1021_MOESM12_ESM.tif]

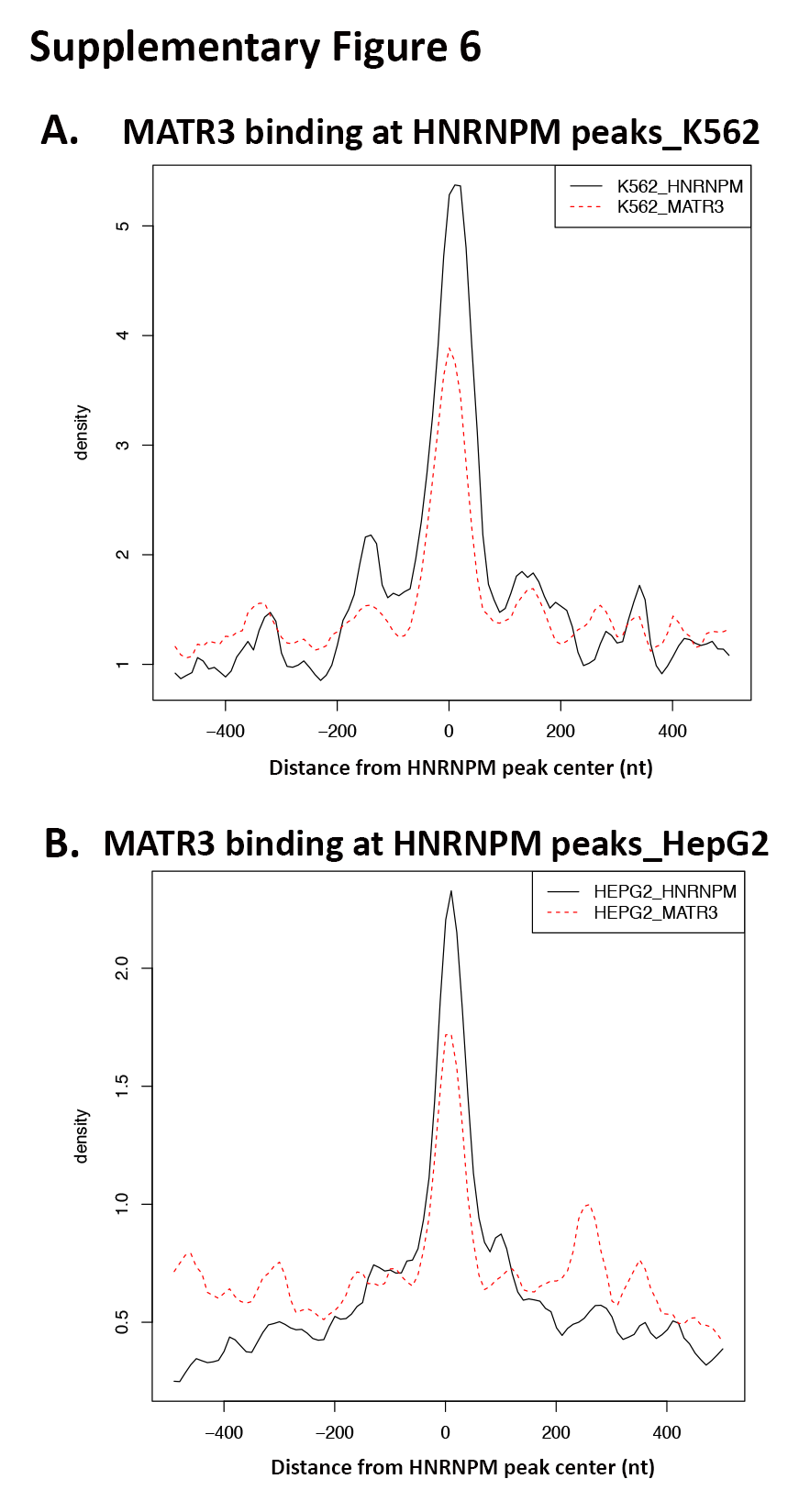

Supplement: Supplementary file 13 — Additional file 13: Figure S6. MATR3 is enriched at hnRNPM eCLIP peaks. (A,B) Read density plots showing normalized read density of MATR3 eCLIP centered at significantly enriched hnRNPM eCLIP peaks in (A) K562 cells and in (B) HepG2 cells. Read densities were normalized to a ± 400 nucleotide (nt) window around hnRNPM peak center. [file 40478_2020_1021_MOESM13_ESM.tif]

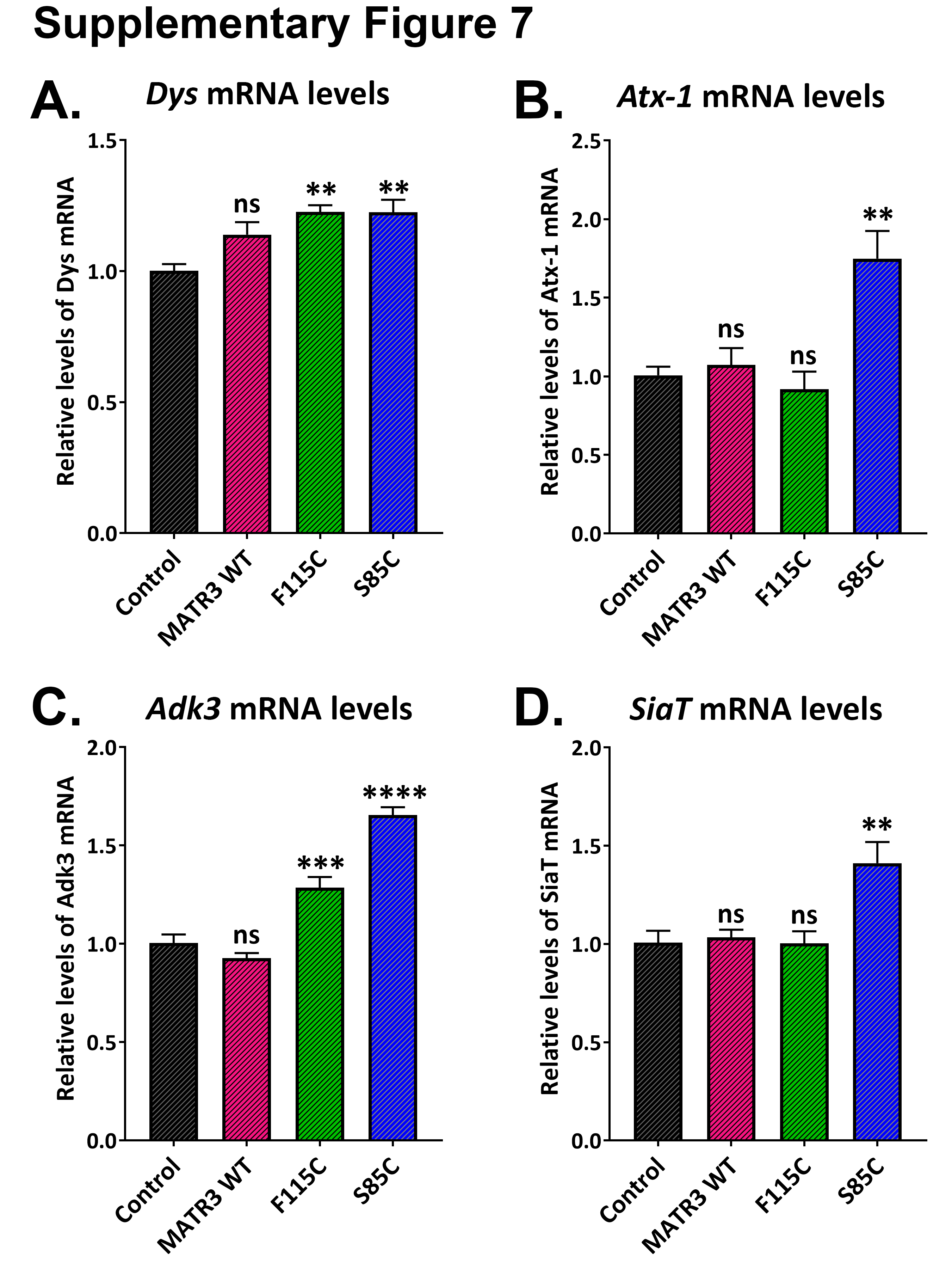

Supplement: Supplementary file 14 — Additional file 14: Figure S7. Levels of candidate targets from MATR3-hnRNPM shared transcriptome altered in Drosophila model. Quantitative graph showing fold change difference in mRNA levels of (A) Dystrophin, Dys, (B) Ataxin-1, Atx-1, (C) Adenylate kinase 3, Adk3, and (D) Sialyltransferase, SiaT, in flies ubiquitously expressing WT and mutant MATR3. mRNA levels of candidate targets are significantly higher in both F115C- and S85C-expressing flies (Dys and Adk3) or only S85C-expressing flies (Atx-1 and SiaT) compared to driver-alone control (n = 5 per group; One-way ANOVA). Error bars indicate S.E.M. **p < 0.01, ***p < 0.001, ****p < 0.0001. [file 40478_2020_1021_MOESM14_ESM.tif]

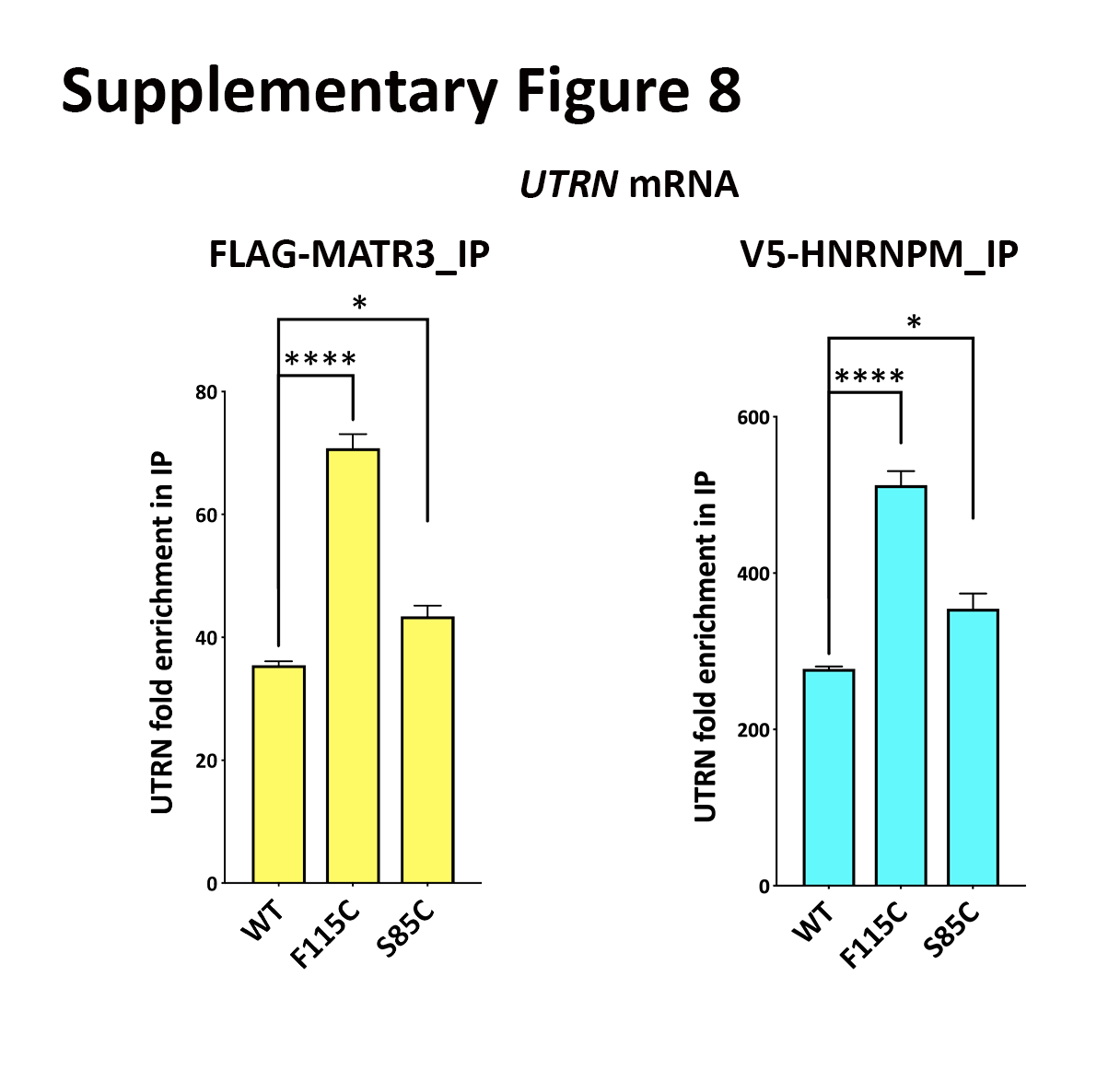

Supplement: Supplementary file 15 — Additional file 15: Figure S8. UTRN mRNA binding to MATR3 and hnRNPM is significantly enriched in cells expressing ALS-causing mutations in MATR3. Fold change differences in UTRN mRNA immunoprecipitated with FLAG-MATR3 and V5-HNRNPM from cells expressing MATR3 WT and ALS-causing mutations F115C and S85C. UTRN binding is significantly enriched to MATR3 (left) and hnRNPM (right) in cells expressing F115C and S85C (n = 3-4 per group; One-way ANOVA). Error bars indicate S.E.M. *p < 0.05, ****p < 0.0001. [file 40478_2020_1021_MOESM15_ESM.tif]
